# Critical roles for WDR72 in calcium transport and matrix protein removal during enamel maturation

**DOI:** 10.1002/mgg3.143

**Published:** 2015-03-29

**Authors:** Shih-Kai Wang, Yuanyuan Hu, Jie Yang, Charles E Smith, Stephanie M Nunez, Amelia S Richardson, Soumya Pal, Andrew C Samann, Jan C-C Hu, James P Simmer

**Affiliations:** 1 Department of Biologic and Materials Sciences, University of Michigan School of Dentistry1210 Eisenhower Pl., Ann Arbor, Michigan, 48108; 2 Department of Pediatric Dentistry, School and Hospital of Stomatology, Peking University22 South Avenue Zhongguancun, Haidian District, Beijing, 100081, China; 3 Facility for Electron Microscopy Research, Department of Anatomy and Cell Biology and Faculty of Dentistry, McGill University3640 University Street, Montreal, Quebec, Canada, H3A 2B2

**Keywords:** Amelogenesis imperfecta, scaffold protein, enamel maturation, hypomaturation, SLC24A4

## Abstract

Defects in *WDR72* (WD repeat-containing protein 72) cause autosomal recessive hypomaturation amelogenesis imperfecta. We generated and characterized *Wdr72*-knockout/*lacZ*-knockin mice to investigate the role of WDR72 in enamel formation. In all analyses, enamel formed by *Wdr72* heterozygous mice was indistinguishable from wild-type enamel. Without WDR72, enamel mineral density increased early during the maturation stage but soon arrested. The null enamel layer was only a tenth as hard as wild-type enamel and underwent rapid attrition following eruption. Despite the failure to further mineralize enamel deposited during the secretory stage, ectopic mineral formed on the enamel surface and penetrated into the overlying soft tissue. While the proteins in the enamel matrix were successfully degraded, the digestion products remained inside the enamel. Interactome analysis of WDR72 protein revealed potential interactions with clathrin-associated proteins and involvement in ameloblastic endocytosis. The maturation stage mandibular incisor enamel did not stain with methyl red, indicating that the enamel did not acidify beneath ruffle-ended ameloblasts. Attachment of maturation ameloblasts to the enamel layer was weakened, and SLC24A4, a critical ameloblast calcium transporter, did not localize appropriately along the ameloblast distal membrane. Fewer blood vessels were observed in the papillary layer supporting ameloblasts. Specific WDR72 expression by maturation stage ameloblasts explained the observation that enamel thickness and rod decussation (established during the secretory stage) are normal in the *Wdr72* null mice. We conclude that WDR72 serves critical functions specifically during the maturation stage of amelogenesis and is required for both protein removal and enamel mineralization.

## Introduction

Dental enamel forms in two major stages, secretory and maturation, defined by the morphology and function of the enamel forming cells, ameloblasts (Reith 1970; Simmer et al. 2010). During the secretory stage, enamel matrix proteins, mainly amelogenin (AMEL), ameloblastin (AMBN), and enamelin (ENAM), are secreted by tall columnar ameloblasts (Fincham and Simmer 1997; Fincham et al. 1999). The initial enamel mineral ribbons are formed and elongate at the mineralization front where these proteins are secreted (Simmer et al. 2012). As the ameloblasts retreat from the dentinoenamel junction (DEJ), a significant amount of protein is deposited in the extracellular matrix, and the enamel layer grows in thickness in tandem with the elongation of the thin mineral ribbons at the enamel surface. The secretory stage enamel is rich in proteins and soft in consistency. Once the enamel reaches its full thickness, the ameloblasts transform into shorter columnar cells and greatly reduce matrix secretion (Kallenbach 1974). After this transition, enamel formation enters the maturation stage. During this stage, the enamel hardness significantly increases by ion deposition onto the sides of the thin enamel crystallites that formed previously during the secretory stage. Crystallite thickening eventually leads to the interlocking of adjacent crystallites, which gives the mature enamel its ultimate hardness (Smith 1998). Ion deposition takes place at the expense of secreted matrix proteins, which are degraded and removed (Smith et al. 1989). MMP20 (matrix metalloproteinase 20) and KLK4 (kallikrein-related peptidase 4) are two proteases shown to be responsible for the degradation of enamel matrix proteins (Lu et al. 2008). The degraded proteins are re-absorbed by maturation stage ameloblasts to provide space for ion deposition, leaving the mature enamel with only a trace residue of its formerly abundant organic matrix.

Disturbances during individual developmental stages of enamel formation lead to distinct defects of dental enamel. Amelogenesis imperfecta (AI) is a group of inherited human diseases with dental enamel defects. While the hypoplastic AI (thin enamel) results from aberrations in appositional growth of enamel at secretory stage, the hypomaturation AI (soft enamel) features defective mineralization and hardening of enamel at maturation stage. Discerning the genetic etiologies of the many different types of AI is helping to identify the molecular participants that are critical for each stage of enamel formation. Mutations in genes encoding secreted enamel matrix proteins (*AMELX,* OMIM *300391; *ENAM*, OMIM *606585; and *AMBN,* OMIM *601259) have been shown to cause hypoplastic AIs, since these genes are mainly expressed and function during the secretory stage. On the other hand, mutations in the two secreted enamel proteases, MMP20 and KLK4, impair degradation of matrix proteins and result in hypomaturation forms of AI. The discovery that mutations in *WDR72* (WD repeat-containing protein 72; OMIM *613214) cause autosomal recessive hypomaturation AI, first demonstrated that this gene is critical for enamel maturation (El-Sayed et al. 2009). While seven different disease-causing *WDR72* mutations have been identified to date (El-Sayed et al. 2009, 2011; Lee et al. 2010; Wright et al. 2011; Kuechler et al. 2012; Katsura et al. 2014), the functions of WDR72 and its role in enamel maturation remain largely unknown. Human *WDR72* encodes an intracellular protein of 1102 amino acids with no known functional domains except a *β*-propeller structure composed of WD40 repeat domains in its N-terminus. Although it is suspected that WDR72 might be involved in vesicle turnover by maturation stage ameloblasts (Katsura et al. 2014), additional experimental evidence is needed before its function can be fully understood.

In this study, we generate *Wdr72* knockout (*Wdr72*^−/−^) mice to investigate the roles of WDR72 in enamel formation and show that the null mice phenocopied the human hypomaturation AI. By characterizing the enamel defects of *Wdr72*^−/−^ mice *in vivo* as well as identifying potential interacting proteins of WDR72 *in vitro*, we demonstrate that WDR72 is necessary for protein re-absorption and calcium excretion by maturation stage ameloblasts. Our findings not only unravel the cellular functions of WDR72, but also gain additional insights into the molecular mechanisms of enamel maturation.

## Materials and Methods

All procedures involving animals were reviewed and approved by the IACUC committee at the University of Michigan.

### Generation of knockin mice

The knockin construct was designed and the mice (species: *Mus musculus,* strain C57BL/6) were generated by GenOway, (Lyon, France). The generation of knockin (KI) gene precisely replaced the *Wdr72* coding sequence in exon 2 with the coding sequence of a *lacZ* (*β*-galactosidase) reporter containing a mouse nuclear localization signal (NLS) upstream of a phosphoglycerine kinase (PGK) promoter driving a neomycin (Neo) selection marker. The PGK-Neo coding sequence was flanked by loxP sites and later deleted by Cre recombinase (Cre). The deleted *Wdr72* sequence and the insert sequence are provided in Figure S1.

### Physical assessment and photography

Heterozygous mutant (*Wdr72*^+/−^) mice were mated to generate three mouse *Wdr72* genotypes (+/+, +/−, and −/−), which were then evaluated for their physical appearance, activity, growth rate, size difference, food intake, and fertility. For gross evaluation of the dental enamel, mice were put under anesthesia using isofluorane, and the incisors were inspected under a dissection microscope. Day 14 and 7-week-old mice were sacrificed and fixative-perfused with 4% paraformaldehyde (PFA). The mandibles were removed and sliced through the mental symphysis with a razor blade to generate hemimandibles. These were carefully dissected free of soft tissues under a stereoscopic microscope using tissue forceps and a spoon excavator. The hemimandibles were submerged in 1% NaClO for 5 min, rinsed, air dried, and photographed using a Nikon SMZ1000 dissection microscope equipped with a Nikon digital camera DXM1200 (Melville, NY).

### Whole-mount X-gal staining

Mouse heads were harvested from 5-week mice, cut in half, and fixed with 4% PFA overnight at 4°C. The tissues were then washed with phosphate-buffered saline (PBS) and incubated at 45°C for 3 days in freshly prepared X-gal staining buffer, pH 8.0, containing 1 mg/mL X-gal, 100 mmol/L HEPES (4-(2-Hydroxyethyl)-1-piperazine ethanesulfonic acid), 5 mmol/L potassium ferricyanide, 5 mmol/L potassium ferrocyanide, 1 mmol/L MgCl_2_, 2% Triton X-100, and 1 mmol/L dithiothreitol. After incubation, the half heads were washed with PBS and inspected under a dissection microscope.

### Immunohistochemistry

Day 11 (D11) and Day 14 (D14) mouse maxillae and mandibles were collected and processed as previously described (Wang et al. 2014b). After embedded in paraffin, the tissues were sectioned at 5 *μ*m thickness, and the sections underwent regular immunostaining and imaging processing (Wang et al. 2014b). The primary antibodies used in the study included anti-*β* galactosidase (1:500, ab9361; Abcam, Cambridge, MA) and anti-SLC24A4 (1:100, ab136968; Abcam) antibodies. The secondary antibody was anti-rabbit IgG conjugated with Alexa Fluor 488 (1:500, A11034; Invitrogen, Carlsbad, CA).

### Scanning electron microscopy

Scanning electron microscopy (SEM) evaluations were performed at the University of Michigan Microscopy and Image-analysis Laboratory (Ann Arbor, MI). Ethanol dehydrated, air-dried hemimandibles and mandibular incisors from Day 14 and 7-week-old *Wdr72*^+/+^, *Wdr72*^+/−^ and *Wdr72*^−/−^ mice were mounted on metallic stubs using conductive carbon cement, offgassed in a vacuum desiccator overnight, and sputter coated with an Au–Pd film to increase conductivity. The samples were imaged using an Amray EF 1910 Scanning Electron Microscope operating at an accelerating voltage of 3–5 kV.

### Backscattered scanning electron microscopy

This backscattered scanning electron microscopy (bSEM) procedures were described previously (Smith et al. 2011a). Soft tissue was removed from the left and right hemimandibles of *Wdr72*^+/+^, *Wdr72*^+/−^, and *Wdr72*^−/−^ mice at 7 weeks, and cross-sectioned at 1 mm increments from the basal (growing) end of the incisors, and imaged by bSEM. For whole incisor surface imaging, the bony caps and soft tissue covering the mandibular incisors were carefully removed, and examined at 50× magnification with a Hitachi S-3000N variable pressure scanning electron microscope using the backscatter mode at 25 kV and 20 pascal pressure.

*Wdr72*^+/+^, *Wdr72*^+/−^, and *Wdr72*^−/−^ mouse molars were prepared as follows: The D14 mandibles were submerged in 4% PFA overnight; 7-week-old mice were fixative perfused with 4% PFA. The hemimandibles were carefully dissected of soft tissues under a stereoscopic microscope using tissue forceps and a spoon excavator, submerged in 1% NaClO for 5 min, rinsed, and acetone dehydrated (30%, 50%, 70%, 80%, 90%, 100%). The hemimandibles were mounted on metallic stubs using conductive carbon cement and carbon coated to increase conductivity and examined using a Hitachi (Century City, Los Angeles, CA) S-3000N variable pressure scanning electron microscope using the backscatter mode.

### Histological staining and analyses

#### Incisor histology

*Wdr72*^+/+^, *Wdr72*^+/−^, and *Wdr72*^−/−^ mice at 7 weeks were deeply anesthetized with isoflurane, perfused with 2.5% glutaraldehyde, 0.05% calcium chloride, 0.1 mol/L sodium cacodylate buffer (pH 7.2–7.4), post fixed for 2 h, and rinsed three times for 15 min each with 0.1 mol/L sodium cacodylate buffer, and postfixed for 60 min in 1% osmium tetroxide in same buffer. The samples were decalcified at 4°C by immersion in 1 L of 4.13% disodium ethylenediaminetetraacetic acid (EDTA, pH 7.3) with agitation. The EDTA solution was changed every other day for 30 days. The samples were washed in PBS at 4°C 4–5 times (every 0.5–1 h) followed by one overnight wash, and dehydrated using a gradient of ethanol and xylene, embedded in paraffin, and sectioned at 0.5 *μ*m thickness using a Microm HM355S (Thermo Fisher Scientific Inc., Waltham, MA). The sections were deparaffinized using xylene and ethanol, stained with 0.1% toluidine blue for 30 sec, dehydrated with ethanol and xylene, mounted on slides, and photographed using a Nikon Eclipse E600 equipped with Nikon digital camera DXM1200. Some decalcified incisors were also processed for embedding in Epon and for semi-sectioning as described elsewhere (Smith et al. 2011b).

#### Molar histology

Day 5, 11, and 14 mouse heads were quickly dissected free of skin, cut in half, and immersed in 4% PFA fixative overnight at 4°C, washed in PBS 4–5 times (every 0.5–1 h) at 4°C, and decalcified at 4°C by immersion in 1 L of 4.13% disodium EDTA (pH 7.3) with agitation. The EDTA solution was changed every other day for 8 to 9 days for D5 mice, 19–21 days for D11 mice, and 30 days for D14 mice. The samples were washed in PBS at 4°C 4–5 times (every 0.5–1 h) followed by one overnight wash. The samples were dehydrated using a graded ethanol series followed by xylene, embedded in paraffin, sectioned at 5 *μ*m thickness, spread on a water bath (52°C), loaded on plus gold glass slides (Thermo Fisher Scientific), left to dry at room temperature overnight and stained with hematoxylin and eosin (H&E) stain.

### Micro-indentation hardness evaluation

Three littermates at 7 weeks from each genotype (*Wdr72*^+/+^, *Wdr72*^+/−^, and *Wdr72*^−/−^) were microhardness tested. Hemimandibles were cleaned free of soft tissue and embedded in Epon resin (EMbed812 cat#14120; Electron Microscopy Sciences, Hatfield, PA) following graded acetone dehydration (30%, 50%, 70%, 80%, 90%, 95%, 100% ×2 for 30 min each). Following polymerization at 65°C, the incisors were cross-sectioned with a model 650 low speed diamond wheel saw (South Bay Technology Inc., San Clemente, CA) at the level of the crest of the alveolar bone close to where the incisor erupts into the mouth, about 8 mm from the apex of the incisor. The sectioned hemimandibles were re-embedded in Castolite AC (Eager Polymers, Chicago, IL) using 25-mm SteriForm molds (Struers Inc., Westlake, OH) with the cutting plane face down and allowed to harden overnight. The transversely sectioned faces of the mandibular incisors were polished sequentially using a syntron polisher on 400, 800, and 1200 grit waterproof silicon carbide papers followed by 1 *μ*m diamond polishing paste (South Bay Technology Inc.).

Microhardness testing was performed using a LM247AT microhardness tester (Leco Corp. St. Joseph, MI) with a load of 10 g for 10 sec with a Knoop tip to obtain a Knoop hardness number (KHN). Measurements were made at 500× magnification. Indentations were placed in the outer, middle, and inner enamel as well as the dentin as a control reading for a total of four indentations per row. This series was performed three times in each animal, for a total of twelve points per animal. Outer enamel is defined as 15 *μ*m from the outer surface of the enamel layer. Inner enamel is defined as within 15–20 *μ*m from DEJ on the enamel side. Middle enamel was defined as halfway between the outer and inner enamel indentation, with a range of 50–60 *μ*m from the outer enamel surface. The dentin indentation was placed 20 *μ*m away from the DEJ on the dentin side. Points that landed in cracks or damaged portion of the enamel or dentin were not read. Statistical significance was determined by pairwise *t*-test using SPSS (Statistical Package for the Social Sciences, IBM Corporation, Armonk, NY) software.

### Protein extraction and analyses from mouse molars

The detailed protocol of protein extraction from mouse molars and the analyses were previously described (Yamakoshi et al. 2011). In brief, D14 first molars were harvested and, for 4 molars collected from one mouse, incubated in 1 mL of 0.17 N HCl/0.95% formic acid for 2 h at 4°C. After the undissolved material was removed by centrifugation, the crude protein extract in strong acid was buffer exchanged with 0.01% formic acid using a centrifugal 3K-filter unit (UFC800324; Amicon by EMD Millipore, Billerica, MA). The concentrate containing proteins extracted from 4 mol/L was raised back to 250 *μ*L of 0.01% formic acid and used for subsequent sodium dodecyl sulfate polyacrylamide gel electrophoresis (SDS-PAGE), Coomassie Brilliant Blue (CBB) staining, and amelogenin immunoblotting.

The amount of protein applied per lane for SDS-PAGE was normalized for each genotype on a per-tooth basis. For CBB staining, 3/17 of a tooth was applied per lane; and for immunoblotting, 1/17 of a tooth was used. After transblotting, the membrane was immunostained with polyclonal rabbit anti-full-length mouse recombinant amelogenin antibody (rM179; 1:2000) and then visualized using ECL prime western blotting detection reagent (RPN2232; GE Healthcare Life Sciences, Piscataway, NJ).

### Recombinant mouse WDR72 expression and affinity purification-mass spectrometry

Mouse *Wdr72* open reading frame (ORF) was cloned into pCMV-Tag4A vector (211174; Agilent Technologies, Santa Clara, CA) to express C-terminal FLAG-tagged mouse WDR72. Human HEK293 cells were cultured on a 10-cm Petri dish and transfected with pCMV-Tag4A-m*Wdr72* construct by following standard cell culture and transfection protocol (Wang et al. 2013). Then, 48 h following transfection, the cells were washed twice with cold PBS and lysed with 3 mL NP40 cell lysis buffer (FNN0021, Novex®; Life Technologies, Carlsbad, CA) with 1 mmol/L phenylmethylsulfonyl fluoride (PMSF) (P7626; Sigma-Aldrich, St. Louis, MO) and 1× protease inhibitor cocktail (P2714; Sigma-Aldrich). After 30 min lysis, the lysates were collected and centrifuged at 15,000 rpm for 10 min. The supernatants were then used for immunoprecipitation (affinity purification).

Dynabeads® protein A immunoprecipitation kit (10006D, Novex® by Life Technologies) was used for immunoprecipitation. The experimental procedure followed the protocol provided by the manufacturer. In brief, anti-FLAG antibody (1:20, F7425; Sigma-Aldrich) was incubated with protein A-attached Dynabeads for 30 min. The antibody/Dynabeads complexes were then mixed with the cell lysate and incubated for another 30 min at room temperature. After washed twice, the immunoprecipitates were eluted and assayed for SDS-PAGE and CBB staining. Six specific protein bands were sliced out from the CBB-stained gel and submitted to Keck Biotechnology Resource Laboratory at Yale University, where trypsinization, liquid chromatography and tandem mass spectrometry (LC-MS/MS) mass spectrometry for protein identification, and subsequent data analysis were performed.

### Banding pattern staining of mouse incisors

The staining solution was freshly prepared by adding 200 mg Methyl Red in 90 mL of 95% methanol. Mandibular incisors of 7-week mice were freshly and carefully dissected from bone. The enamel organ epithelium over the enamel (labial) surface was gently wiped off with ice-cold gauze. The incisors were immediately soaked in the staining solution at room temperature for 2 min, air dried for 5 min, and inspected under dissection microscope.

## Results

### *Wdr72*-knockout/*lac*Z-knockin mice generation and dental phenotype

**Figure 1:**
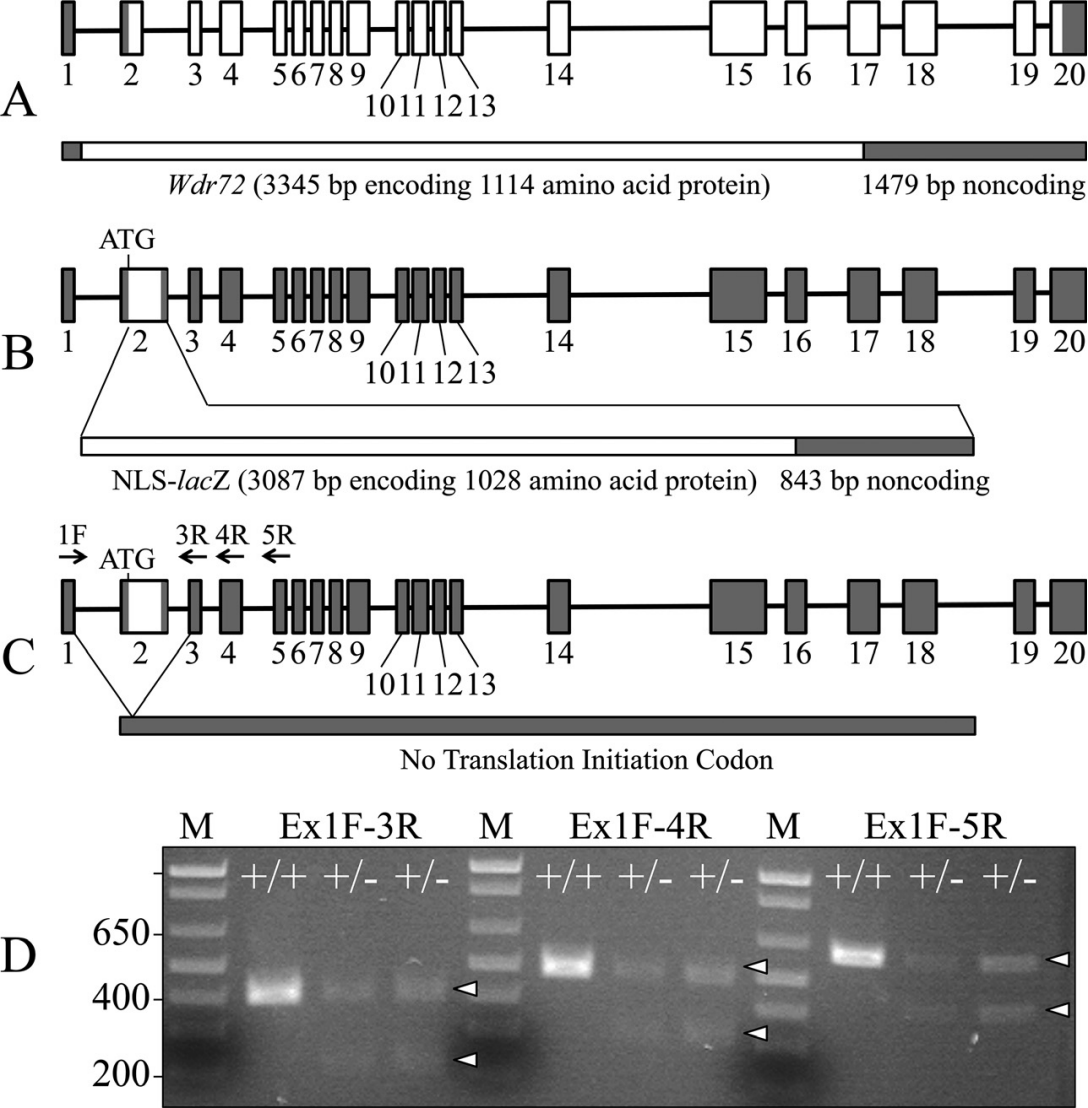
*Wdr72* knockout/NLS-*lac*Z knockin construct and expression. (A) Diagram of the wild-type *Wdr72* gene. This diagram is based upon a comparison of the mouse *Wdr72* NCBI cDNA and genomic reference sequences NM_001033500.3 and NC_000075.6, respectively. Exons are boxes with shaded areas corresponding to noncoding regions. (B) Knockin construct showing replacement of exon 2 starting at the translation initiation codon with NLS-*lac*Z. (C) Alternative splicing of transcripts from the *Wdr72* null allele that read through the transcription termination sequences introduced downstream of the NLS-*lac*Z sequence in intron 2. Arrows identify primer annealing sites for RT-PCR. (D) Ethidium bromide stained agarose gel showing RT-PCR products generated by amplification of RNA isolated from enamel organ epithelia (EOE). DNA sequencing of the bands showed that the modified exon 2 was sometimes deleted during RNA splicing. The higher bands marked by arrowheads are the wild-type amplification products; the lower bands marked by arrowheads are the same products lacking exon 2.

To investigate the role of WDR72 in enamel formation *in vivo*, we used gene targeting to ablate *Wdr72* in mouse strain C57Bl6 (Fig.1 and Fig. S1). Mouse *Wdr72* has 20 exons, with the first exon being noncoding. We replaced the coding sequence of exon 2 and the 5′ end of intron 2 with a *lacZ* reporter gene encoding *β*-galactosidase fused to a mouse NLS. The translation initiation codon (ATG) of the inserted NLS-*lacZ* exactly replaced that of *Wdr72*, and thus precluded translation of native WDR72 and established a reporter gene in an optimal context to mimic *Wdr72* transcription *in vivo*. The NLS-*lacZ* cassette included the coding sequence for bacterial *β*-galactosidase followed by a 3′-untranslated region (UTR) containing two polyadenylation signals to terminate transcription in intron 2.

**Figure 2:**
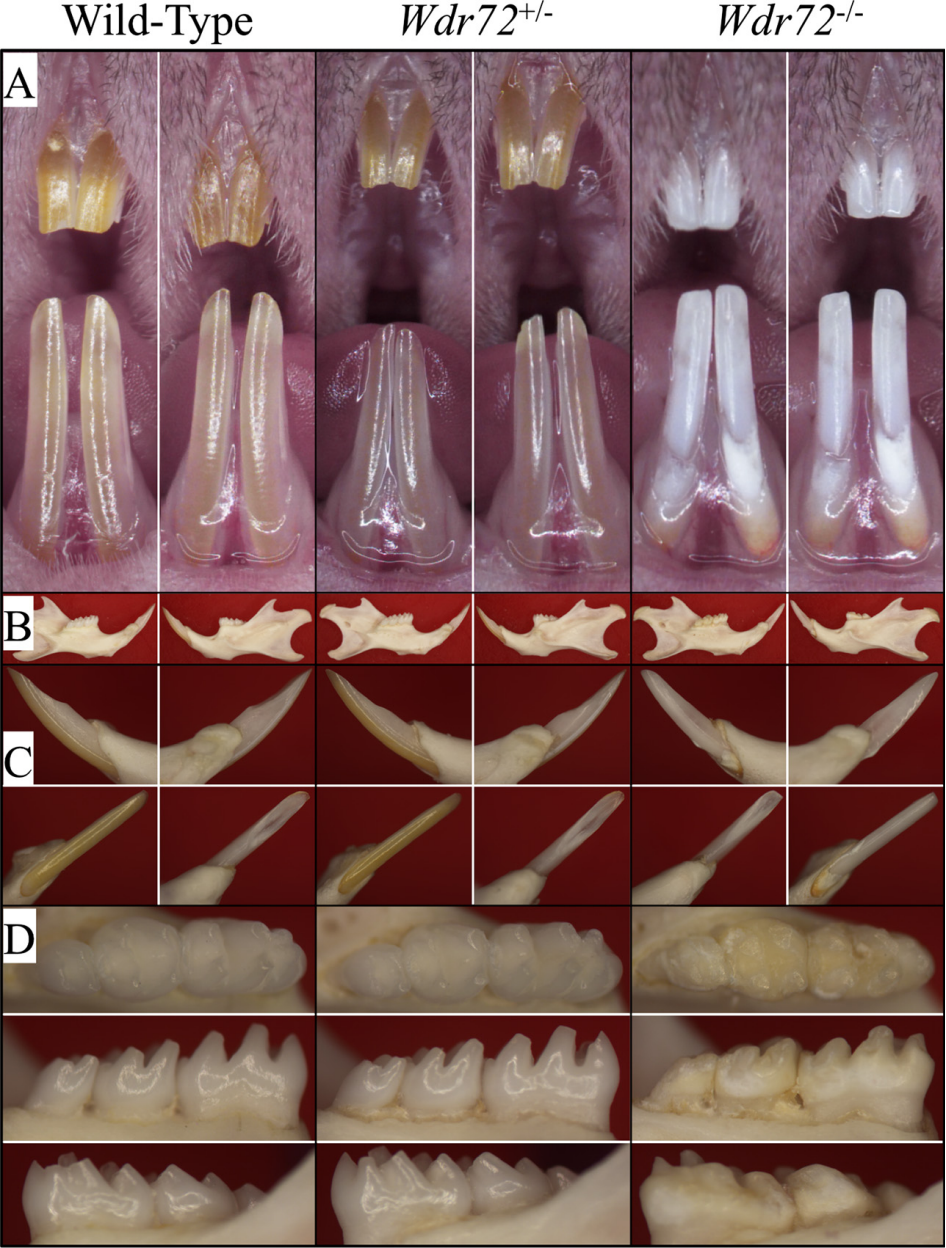
Photographs of 7-week-old mice of three *Wdr72* genotypes. (A) Frontal view of maxillary and mandibular incisors. The null incisors exhibited a chalky-white appearance and chipped incisal edges. (B) Medial (left) and lateral (right) views of hemimandibles. The size and the bony structures of the mandible in null mice appeared normal and comparable to those of the wild-type and heterozygous mice. (C) Lateral (upper left), medial (upper right), labial (lower left), and lingual (lower right) views of mandibular incisors (D) Occlusal (upper), lingual (middle), and buccal (lower) views of mandibular molars. The *Wdr72*^*−/−*^ molars showed extensive attrition with dull, yellowish areas of exposed dentin.

The knockout (*Wdr72*^−/−^) mice could be readily identified by their enamel defects, which included conspicuous chalky-white, chipped incisors (Fig.2). The molar enamel also suffered severe attrition, even though the animals were provided soft chow. Enamel malformations were apparent on the molars at D14, even prior to their eruption (Fig. S2). Conspicuous surface defects in the erupting D14 null molars were sometimes generated while removing the overlying soft tissue to expose the crowns for inspection. Enamel peeled off the null molars, highlighting major structural weaknesses in the *Wdr72*^−/−^ enamel layer. Heterozygous (*Wdr72*^+/−^) mouse enamel was indistinguishable from that of the wild-type (Fig.2 and Fig. S2). Mating the heterozygous mice yielded the expected Mendelian (1:2:1) ratio, and mice of all genotypes were generally healthy, viable, and fertile.

### *β*-galactosidase expression in maturation stage ameloblasts

Whole-mount X-gal staining of 5-week-old skinned *Wdr72*^+/−^ mouse heads showed positive histostaining along the labial surfaces of the incisors, specifically in the region corresponding to maturation stage of enamel formation (Fig. S3). This specificity was confirmed by *β*-galactosidase immunohistochemistry of mouse mandibular incisor sections. While no immunoreactivity was detected in secretory stage ameloblasts, positive signal was evident in the nuclei of maturation stage ameloblasts (NLS-*lac*Z product), indicating a specific expression of *Wdr72* during the maturation stage, rather than the secretory stage (Fig. S4). Despite the clear result, signal for *Wdr72* expression was weaker than expected. We performed RT-PCR of enamel organ epithelia to confirm expression of the wild-type *Wdr72* gene and the targeted knockin gene in heterozygous (*Wdr72*^+/−^) mice. In addition to the wild-type *Wdr72* transcript, an alternatively spliced transcript joining exon 1 with exon 3 (skipping the knockin exon 2) was also expressed (Fig.1D). This finding suggested that NLS-*lacZ* was frequently deleted during RNA splicing and explained the weak X-gal staining in the genetically engineered mice.

### Hypomaturation enamel defects in *Wdr72* null mice

In general, the *Wdr72* null mice (*Wdr72*^−/−^) looked grossly normal and similar to their wild-type (*Wdr72*^+/+^) and heterozygous (*Wdr72*^+/−^) littermates. However, in contrast to the yellow/brown color of normal murine incisor enamel, all incisors of 7-week-old *Wdr72*^−/−^ mice exhibited chalky-white labial enamel (Fig.2). Also, the tips of null incisors appeared to be chipped and rounded, and lacked the pointed sharpness of wild-type incisors.

**Figure 3:**
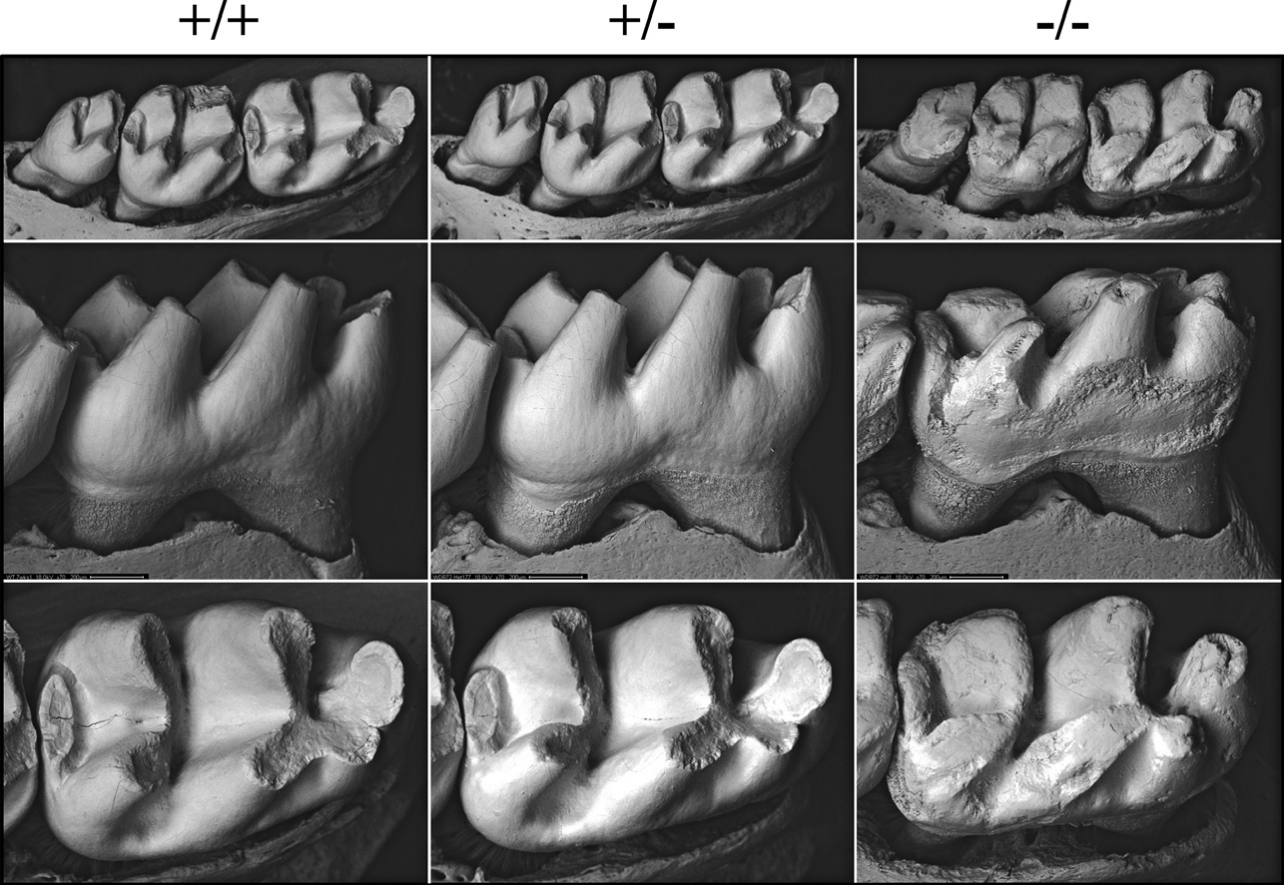
Backscattered SEMs of 7-week mouse mandibular first molars. The top panels show occlusal views; the middle panels show lingual views; the bottom panels show occlusal views. Although their basic morphologies stayed the same, all *Wdr72*^*−/−*^ molars exhibited severe attrition, leaving blunted cusps and rough enamel surfaces.

**Figure 4:**
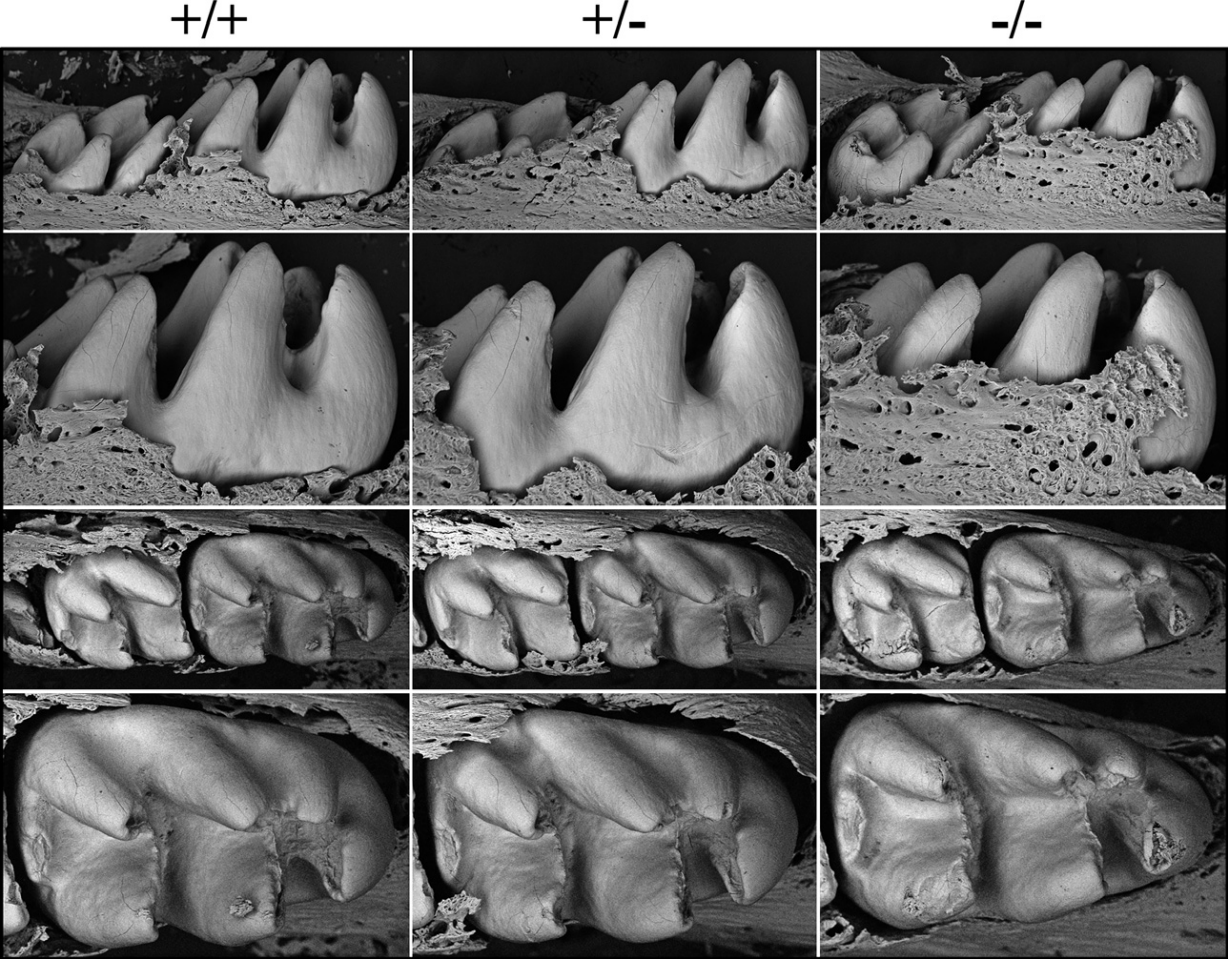
Backscattered SEMs of Day 14 *Wdr72* mouse mandibular molars following soft tissue removal. The top two rows are lingual views; the bottom two rows are occlusal views. The sizes of the teeth and the thicknesses of the enamel appeared to be similar in the three genotypes. These images show the molars prior to eruption. Immediately after eruption the enamel layer is abraded away.

We examined the labial surfaces of 7-week mouse mandibular incisors by bSEM. The wild-type and heterozygous incisors had smooth enamel surfaces, while the enamel of null incisors showed surface roughnesses unavoidably inflicted during soft tissue removal and due to severe attrition incisally (Fig. S5). Attrition was also observed on 7-week *Wdr72*^*−/−*^ molars inspected by bSEM (Fig.3), which was not the case for the D14 null (unerupted) molars that exhibited relatively normal crown size and shape (Fig.4).

**Figure 5:**
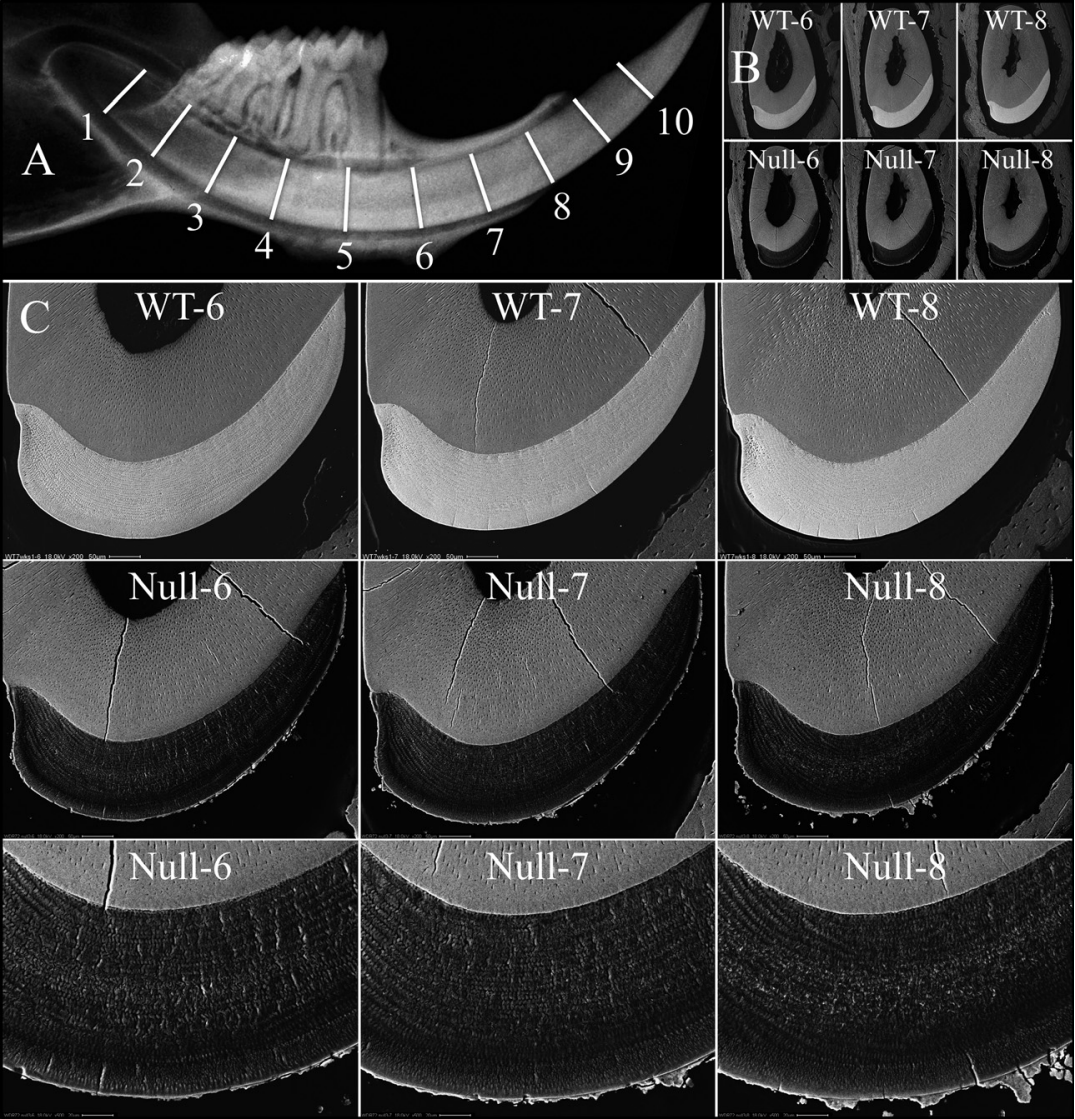
Backscattered SEMs of 7-week mandibular incisor cross-sections from late maturation stage. (A) Hemimandible radiograph showing the 1 mm-incremental cross-section levels from apical to incisal along a mandibular incisor. (B) Low-magnification views of mandibular cross-sections at levels 6–8, of wild-type (top) and null (bottom) incisors. (C) Higher magnification bSEM images of level 6–8 cross-sections. The electron densities of the *Wdr72*^*−/−*^ enamel at all three levels were similar to each other and significantly lower than those of the wild-type enamel (upper panel), demonstrating a severe hypomineralization defect of the *Wdr72*^*−/−*^ enamel. Also, an irregular layer of electron-dense crust covered the enamel of the null mice. The complete panel of cross-sections (levels 2–8) for the three genotypes is presented in Fig. S6. bSEM, backscattered scanning electron microscopy.

We assessed the progression of enamel mineralization in the three genotypes (*Wdr72*^+/+^, *Wdr72*^+/−^ and *Wdr72*^−/−^) throughout the multi-stage process of amelogenesis by bSEM analyses of sequential cross-sections at 1 mm increments (from level 2 to 8) along the continuously growing mandibular incisors (Fig.5 and Fig. S6) (Smith and Nanci 1989). In the enamel of the wild-type incisor, the electron density gradually increased as the cross-sections advanced toward the incisal tip, reflecting the progressive increase in mineralization. Significant increases in electron density occurred throughout the maturation stage (levels 4–8). While the enamel of heterozygous (*Wdr72*^+/−^) incisors showed similar electron density patterns as the wild-type, *Wdr72* null incisors exhibited a dramatic reduction in enamel electron density relative to the wild-type, demonstrating severe hypomineralization (hypomaturation) of the enamel in *Wdr72* null mice. Despite the reduced level of mineralization, the full thickness of enamel and the organization of enamel rods in the null incisor were unaffected. The electron density of the null enamel increased initially but then stayed the same in the levels 5–8 cross-sections, indicating that the enamel defects of *Wdr72* null mice arose from aberrations occurring during the maturation (as opposed to secretory) stage of amelogenesis. Furthermore, an irregular layer of electron-dense material covered the surface of the hypomineralized enamel, starting at level 5 and increasing through level 8 (Fig.5 and Fig. S6C). Apparently calcium and phosphate ions released by maturation ameloblasts did not penetrate into the enamel layer, but instead deposited as mineral on the enamel surface, forming a crust with irregular projections radiating into the overlying enamel organ epithelia.

**Figure 6:**
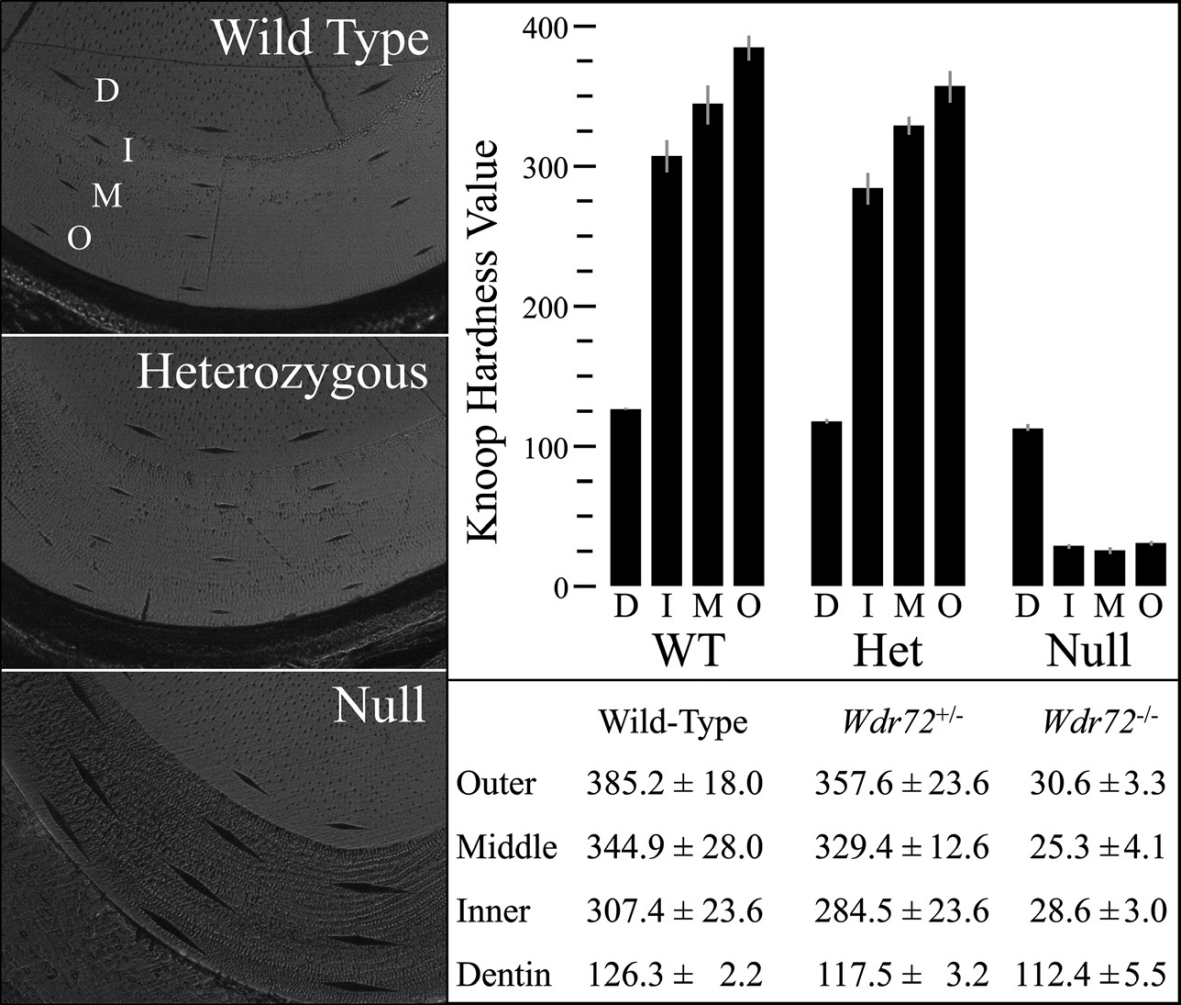
Knoop hardness testing of 7-week mandibular incisors. The left panel shows indentations made by a 10-gm force application in incisor cross-sections at the level of alveolar crest (level 8) for the three genotypes. The hardnesses of the dentin (D), and the outer (O), middle (M), and inner (I) enamel were evaluated. Three indentations were generated at each sample level and measured to calculate the Knoop hardness values (HKs). The right lower panel demonstrates the mean HKs over different areas of different genotypes, and the comparative histograms are presented at the right upper panel. In the wild-type and heterozygous mutant, the HKs of enamel gradually increased from inner to outer areas and are averaged 2.7-fold greater than the measured HK of dentin. In contrast, The *Wdr72*^*−/−*^ enamel exhibited significantly reduced HK values, which averaged only 25% of those for dentin and 10% of those for wild-type enamel.

To evaluate the effects of enamel hypomineralization on the physical properties of *Wdr72*^−/−^ enamel, we performed Knoop hardness testing (KHT) of dentin, the inner enamel (near dentin), the middle enamel, and outer enamel (near the surface) of incisors sectioned at the level of the alveolar crest (level 8) for each of the three genotypes (Fig.6 and Fig. S7). The mean Knoop hardness values (HKs) for enamel were comparable in the wild-type and *Wdr72*^+/−^ mice, while the HKs of *Wdr72*^−/−^ enamel were more than 10 times lower. Also, unlike the gradually increasing HKs from the inner to outer enamel in wild-type and heterozygous mice, the *Wdr72* null enamel exhibited equally low HKs at all three depths (Fig.6). The HKs values for dentin were similar for all genotypes. The HKs for the wild-type and heterozygous enamel averaged 2.7-fold higher than those of dentin, while the HKs of *Wdr72*^−/−^ enamel averaged only a quarter of those for dentin. These results demonstrated a severe enamel hardness (hypomineralization/hypomaturation) defect in *Wdr72* null mice.

### Excess residual enamel proteins after the maturation stage in *Wdr72* null mice

**Figure 7:**
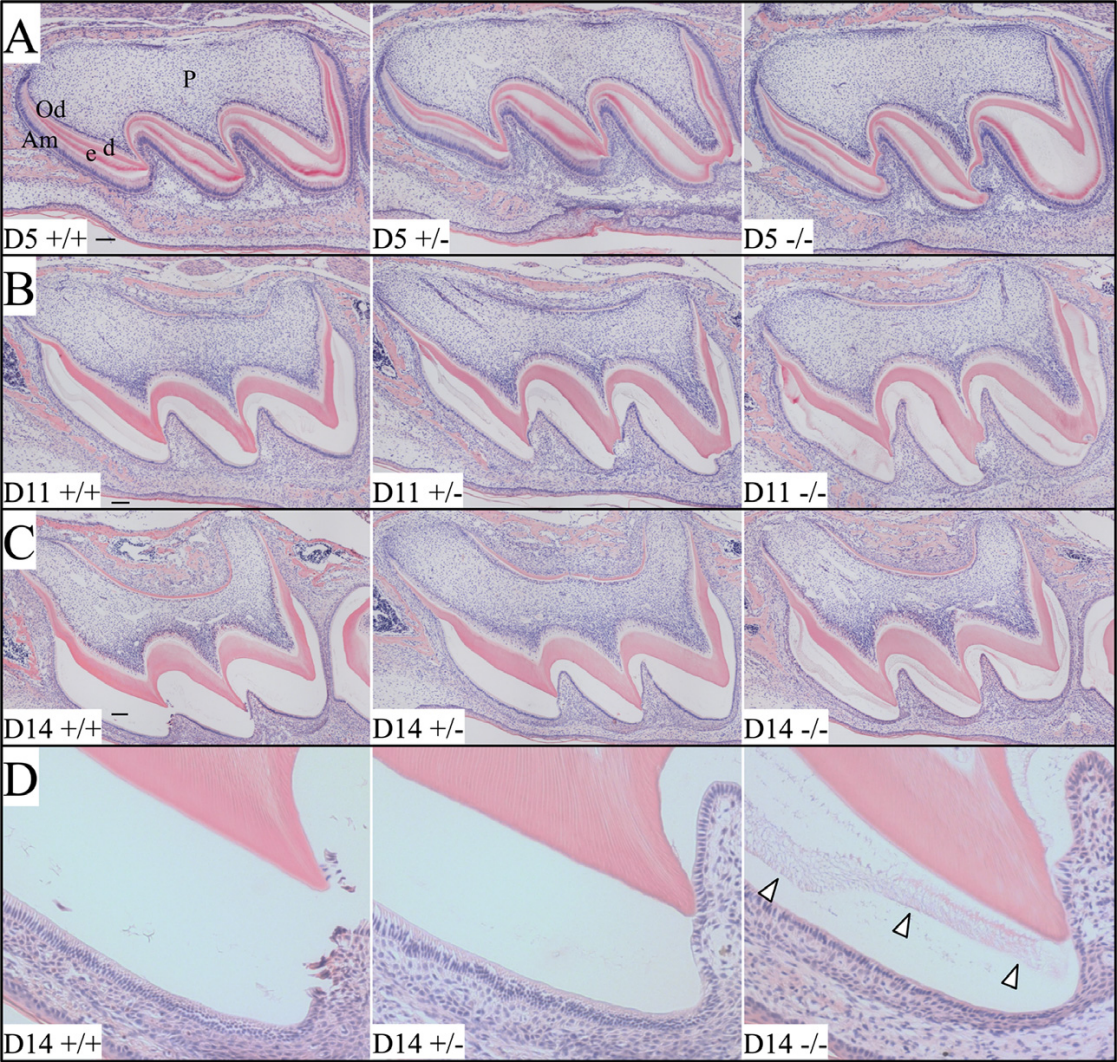
Histology of D5, D11, and D14 maxillary first molars. (A–C) Low-magnification (10×) views of the D5, D11, and D14 maxillary first molars. (D) Higher magnification (20×) views of the D14 first molar mesial cusp detailing residual matrix (arrowheads) in the null molar. At D5, enamel matrices are actively being secreted by ameloblasts and show eosinophilic staining. No evident histological differences could be identified between the first molars of the three genotypes at D5. At D11, reabsorption of extracellular matrix protein was most advanced in the wild-type and heterozygous mice. Residual protein was greatly diminished near the cusp tips where enamel maturation first commenced. By D14 (immediately prior to eruption), most of the protein in the extracellular matrices had been reabsorbed in the wild-type and heterozygous molars, while the null molar retained eosinophilic material in the enamel space (arrowheads). Am, ameloblasts; d, dentin; e, enamel; Od, odontoblasts; P, dental pulp. Scale bars: 100 *μ*m.

To investigate the causes of enamel defects in *Wdr72*^−/−^ mice, we analyzed the histology of the developing maxillary molars at postnatal D5, D11, and D14 (Fig7 and Figs. S8–S10). Ameloblasts in the first molars from these ages were in progressively later stages of development: secretory (D5), mid-maturation (D11), and late maturation (D14) stages. Secretory stage (D5) ameloblasts exhibited a typical tall columnar morphology, and the D5 eosinophilic enamel extracellular matrices appeared similar in all three genotypes (*Wdr72*^+/+^, *Wdr72*^+/−^, and *Wdr72*^−/−^), suggesting that enamel matrix secretion was not affected in the *Wdr72* null mice (Fig.7 and Fig. S8). The mid-maturation stage enamel layers of wild-type and *Wdr72*^+/−^ D11 first molars showed a small amount of residual extracellular matrix at the fossae and cervical areas, while in the null the residual matrix could also be found around cusp tips (Fig.7 and Fig. S9). Residual enamel matrix was abundant in the enamel matrix of *Wdr72*^−/−^ first molars during late maturation (immediately preceding eruption), whereas the wild-type and *Wdr72*^+/−^ molars were virtually empty of enamel protein (Fig.7 and Fig. S10). These results demonstrated that absence of WDR72 leads to impaired or delayed protein matrix removal during enamel maturation.

**Figure 8:**
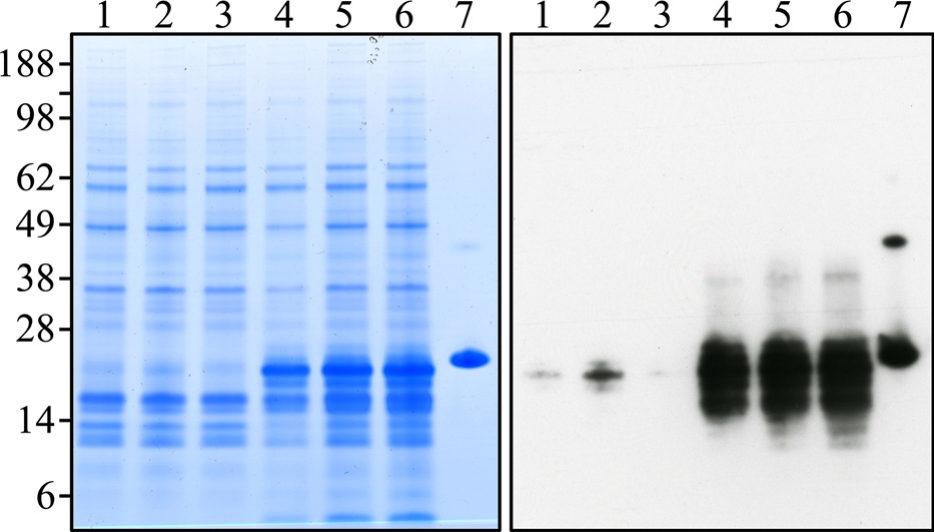
Residual extracellular proteins from D14 mouse first molars. *Left*: polyacrylamide gel stained with CBB. Each lane contains ∼18% of total protein extracted from a single molar. *Right*: transblot from a replica polyacrylamide gel to the one on the left, but each lane contained only 6% of the protein extracted from a single tooth. Sample genotypes: lane 1: *Wdr72*^*+/+*^; lanes 2–3: *Wdr72*^*+/−*^; lanes 4–6: *Wdr72* null; lane 7: rM179 (recombinant mouse amelogenin expressed in bacteria). The apparent molecular weights (in kDa) are shown on the left. The *Wdr72*^*−/−*^ molars had significantly increased residual enamel matrix proteins (like amelogenin) than did the wild-type and *Wdr72*^*+/−*^ molars. The amelogenins in the *Wdr72*^*−/−*^ molars had lower molecular weights than the full-length rM179, indicating that the retained proteins were largely comprised of amelogenin degradation products.

To confirm the histological impression of excess residual proteins in *Wdr72*^−/−^ enamel, we extracted and analyzed proteins from the first molars of D14 mice. Whole first molars were harvested from littermates of three genotypes (*Wdr72*^+/+^, *Wdr72*^+/−^, and *Wdr72*^−/−^), stripped of soft tissues, and rapidly demineralized in acid to release the residual matrix proteins. The crude protein extracts were analyzed by SDS-PAGE and by western blot analysis using an antibody raised against recombinant mouse amelogenin (rM179) (Simmer et al. 1994) (Fig.8). SDS-PAGE detected abundant residual protein in the *Wdr72*^−/−^ molars, relative to those of wild-type and heterozygous mice, and western blot analysis showed that the *Wdr72* D14 null molars contained significantly more amelogenin than the wild-type and *Wdr72*^+/−^ molars. Combined with the histological findings, these results demonstrated an excess of residual enamel proteins in *Wdr72*^−/−^ enamel and suggested that WDR72 plays a critical role in matrix removal during enamel maturation.

### Morphological alterations of maturation stage ameloblasts in *Wdr72* null mice

**Figure 9:**
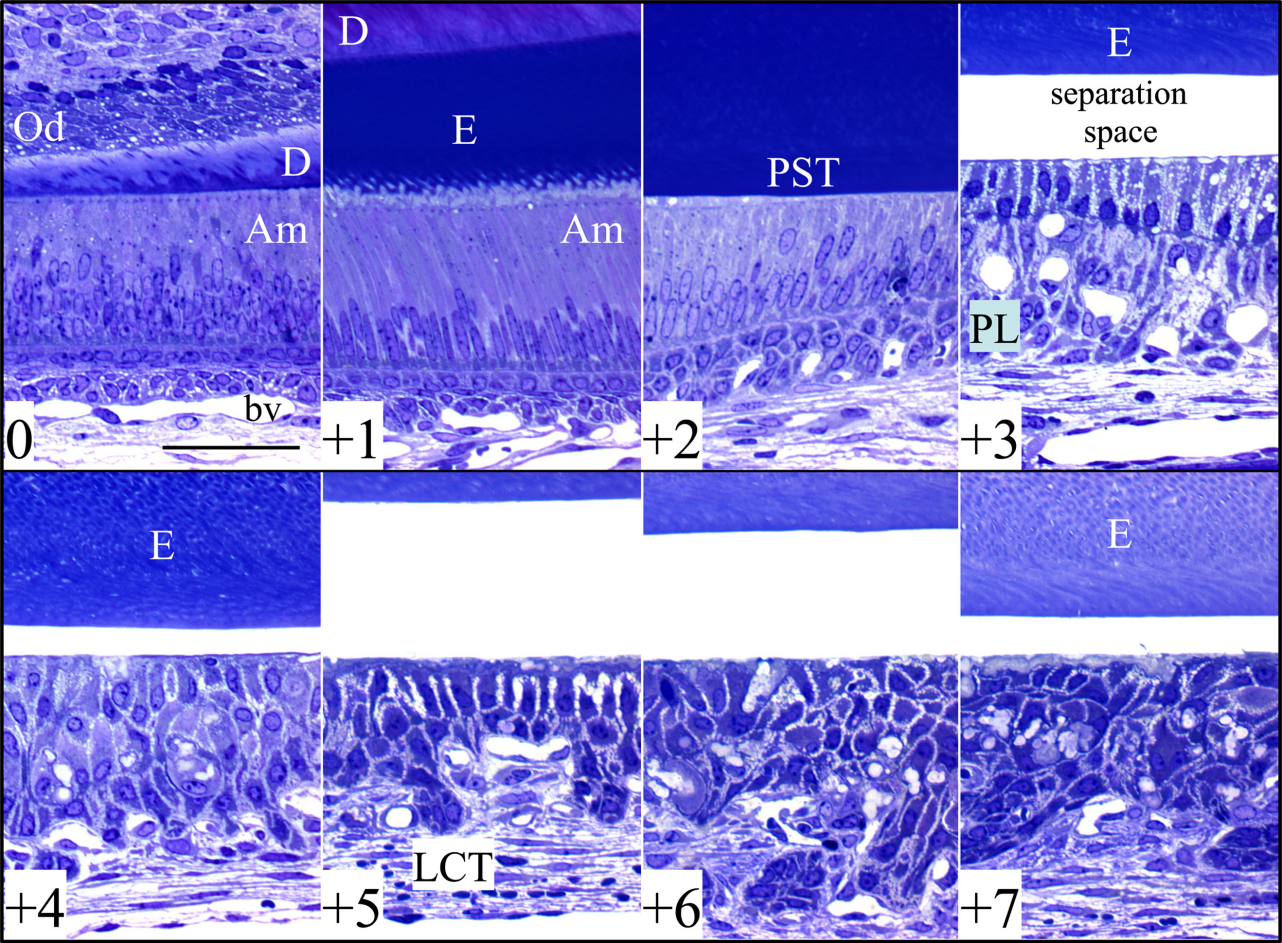
Histology of a 7-week *Wdr72*^*−/−*^ mandibular incisor. The sequential panels show high-magnification views (40×) of a toluidine blue-stained longitudinal Epon section of a *Wdr72* null mandibular incisor taken at 1 mm increments. The unabridged incisor histology is presented in Fig. S11b. The upper left panel (0) shows the initiation of enamel formation near the apical (cervical) end of the incisor. The +1 panel shows secretory stage ameloblasts at a point 1 mm incisally from the previous panel. The +2 panel shows postsecretory transition (PST). Panels +3 to +7 reveal changes from early to late maturation. A separation space between the maturation stage ameloblasts and the enamel was observed in all four longitudinal sections of *Wdr72* null incisors, but not in similarly processed wild-type incisors (Fig. S11a). Although the space between maturation ameloblasts and the enamel surface is likely artifactual, it demonstrates a weakness in the attachment of maturation stage ameloblasts to the enamel surface. The residual enamel matrix persists until eruption but diminishes in staining intensity, suggesting there is only a small reduction in the amount of organic matrix during the maturation stage. The ameloblast layer becomes progressively more disorganized incisally (sections +4 through +7). Am, ameloblasts; bv, blood vessel; D, dentin; E, enamel; LCT, labial connective tissue; Od, odontoblasts; PST, postsecretory transition; PL, papillary layer. Scale bars: 50 *μ*m.

**Figure 10:**
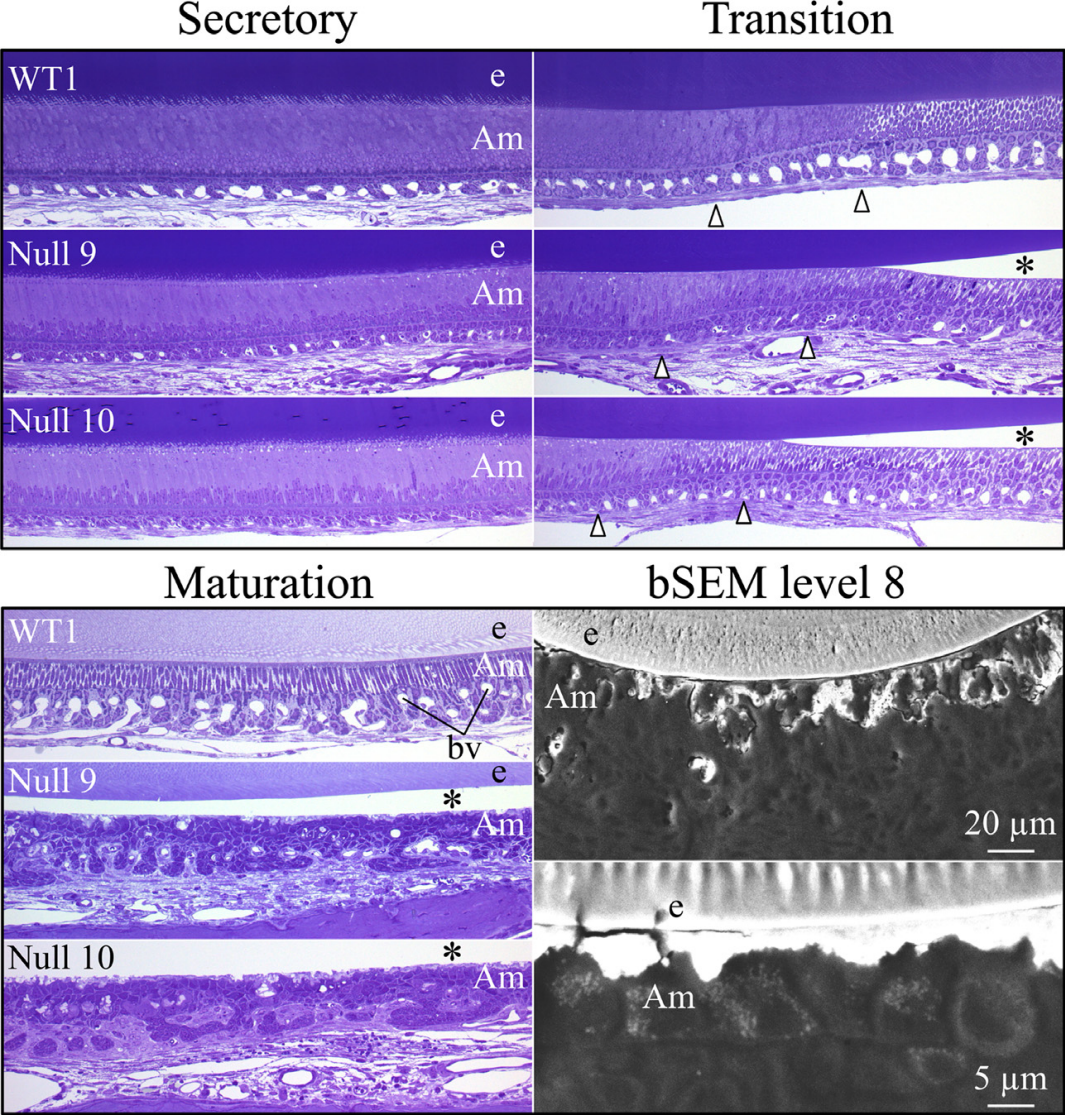
Comparison of wild-type and *Wdr72*^*−/−*^ stages of amelogenesis in 7-week-old mandibular incisors. The wild-type sections are on top. In the *Wdr72*^*−/−*^ *incisors,* the presecretory and secretory stage ameloblasts and the secretory stage enamel layer look normal. Maturation stage ameloblasts detached from the enamel surface during sectioning. The unstained space between the maturation ameloblasts and the enamel surface is marked by an asterisk. Postsecretory transition is delineated by arrowheads. Maturation stage ameloblasts and the papillary layer show signs of disorganization, mucoid deterioration and ectopic mineralization. The papillary layer is dystropic and is associated with fewer blood vessels than in the wild type. Structures along the distal membrane of maturation ameloblasts relate to the pathological mineral crust that forms on the enamel surface. bSEM images show that the mineralization crust is intimately associated with maturation ameloblasts. Am, ameloblasts; bv, blood vessel; e, enamel; bSEM, backscattered scanning electron microscopy.

The histology of longitudinal sections of four *Wdr72*^−/−^ mandibular incisors at 7 weeks revealed no defects during early or middle secretory stage of enamel formation compared with wild-type incisors (Figs.9, 10, and Fig. S11A–E). In all four *Wdr72*^−/−^ incisors examined, maturation stage ameloblasts were separated from the enamel surface (Fig. S11B–E). However, the bSEM images of un-decalcified *Wdr72*^−/−^ incisor cross-section revealed that the maturation stage ameloblasts were intimately associated with the mineral surface, suggesting that the separation is likely the result of a sectioning artifact. Nevertheless, this artifact is not generally observed in wild-type incisors (Fig. S11A) and suggested that *Wdr72*^−/−^ maturation stage ameloblasts are not as tightly adherent to the enamel surface as those of the wild type. *Wdr72*^−/−^ maturation stage ameloblasts and papillary layer cells appear to be disorganized (Fig.9). The residual enamel matrix in the *Wdr72*^−/−^ molars remained present across the maturation stage to the gingival margin (eruption), but gradually diminished in staining intensity, suggesting that some loss of organic material occurs despite significant alterations in the normal organization of the enamel organ (Fig. S11B–E). Multiple amorphous, lightly stained loci were found to locate between maturation ameloblasts, specifically in the later stages of enamel formation, and appeared to disrupt the palisading pattern of ameloblasts (Fig.9). These loci were presumed to be the mineral projections observed on the *Wdr72*^−/−^ enamel surface by bSEM (Fig.5). Finally, blood vessels were noticeably reduced in number and size in the enamel organ across the maturation stage in *Wdr72*^−/−^ incisors (Fig. S11B–E).

### Altered subcellular localization of NCKX4 in *Wdr72*^*−/−*^ ameloblasts

**Figure 11:**
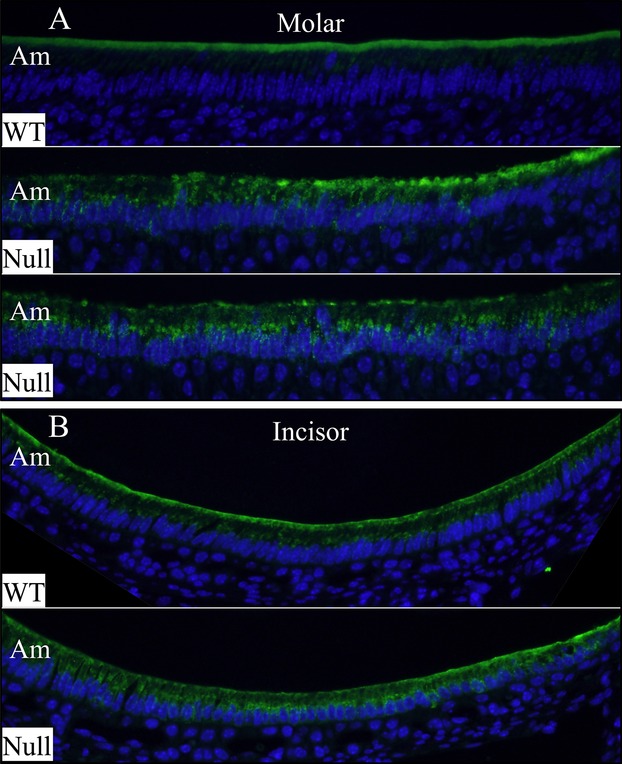
Immunolocalization of NCKX4 in maturation stage ameloblasts. (A) ameloblasts of D11 maxillary first molars exhibited distinct subcellular localizations of NCKX4 in wild-type and null mice. While the wild-type ameloblasts showed strong NCKX4 immunoreactivity at their distal membranes, the NCKX4 signal was detected in the cytoplasm of null ameloblasts, especially in the supranuclear area. (B) In wild-type mice, maturation stage ameloblasts of D14 mandibular incisor exhibited generally strong NCKX4 signals at the distal membrane and weak in the cytoplasm. In contrast, a stronger intracellular NCKX4 immunostaining with weaker signal at distal membrane was observed in some *Wdr72* null ameloblasts. The unabridged molar and incisor immunolocalizations of NCKX4 are presented in Figures S12–13. Am, ameloblasts.

Sodium/potassium/calcium exchanger 4 (NCKX4) encoded by *SLC24A4* is critical for enamel maturation (Parry et al. 2013) and is thought to secrete calcium into the maturation stage enamel matrix (Wang et al. 2014a). To assess if the hypomaturation defects in the enamel of *Wdr72*^*−/−*^ mice can be caused by impaired expression or localization of this calcium transporter, we compared the immunolocalizations of NCKX4 in D11 maxillary molars and in D14 mandibular incisor cross-sections (Fig.11 and Figs. S12–S14). In wild-type mice, specific NCKX4 immunoreactivity was highly concentrated along the distal membranes of maturation stage ameloblasts in molars (Fig. S12) and incisors (Fig. S14), forming a strong fluorescent stripe adjacent to the enamel layer. However, in *Wdr72*^*−/−*^ mice, in addition to a distal membrane localization in some areas, the expression of NCKX4 in molar ameloblasts (Fig. S13) also showed an intracellular dotty pattern, concentrating in the supranuclear area, rather than distal membrane. This distinction was evident, but less apparent in the incisor maturation ameloblasts (Fig. S14), suggesting that the subcellular localization of NCKX4 is altered when WDR72 is absent. The altered NCKX4 localization is not a consequence of ameloblast disorganization, which is minimal in the developing molars and incisors of suckling mice compared to incisor ameloblasts at 7 weeks when the continuously growing incisors may be affected by mechanical stresses associated with function.

### Loss of enamel banding patterns stained by pH indicator in *Wdr72*^*−/−*^ incisors

**Figure 12:**
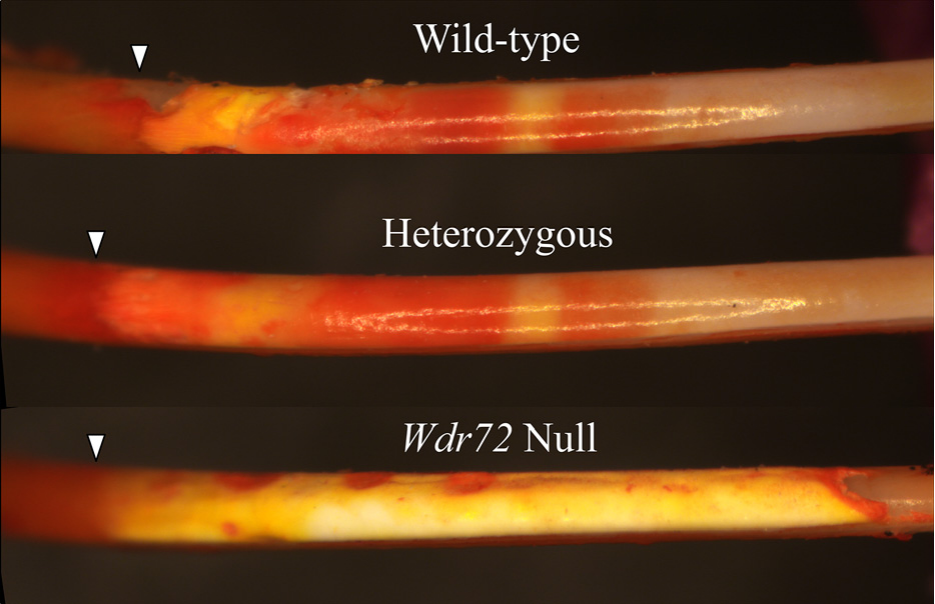
Methyl Red staining pattern of 7-week mandibular incisors. Labial views of the incisors oriented with the apical end toward the left and the incisal end toward the right. In all three genotypes, the secretory zone (left to the arrowheads) stained dark orange. In the maturation zone (right of arrowheads), the incisor enamel surfaces of wild-type and heterozygous mice showed bright orange-red staining interrupted by at least two bands of light yellow staining, while the null incisor exhibited no alternating banding pattern, but instead exhibited a heterogeneous yellowish staining over the rough enamel surface. Arrowheads mark the transition from secretory stage to maturation stage.

It has been demonstrated that characteristic banding patterns could be visualized at the surfaces of rat and mouse incisors in the maturation zone of amelogenesis by staining with certain histochemical and pH indicating stains (Smith et al. 1987; McKee and Warshawsky 1989). Although the exact chemical mechanisms of these stainings are still unknown, it is considered that the banding pattern corresponds to the ameloblast modulation during enamel maturation (Sasaki et al. 1991; Smith 1998). Knowing that *Wdr72*^*−/−*^ enamel had severe hypomaturation defects, we examined 7-week mouse mandibular incisors to see if this banding pattern was altered in *Wdr72*^*−/−*^ mice. Methyl Red pH indicator staining of the wild-type and *Wdr72*^*+/−*^ incisors showed an alternating pattern of orange-red and light yellow bands in the maturation zone, with the staining fading out toward the incisal end (Fig.12). The wild-type pattern showed the normal pattern of pH oscillations over the enamel surface during enamel maturation. The null incisor exhibited no alternating pattern but a heterogeneous yellowish staining over the rough enamel surface, revealing that the normal drop in matrix pH beneath ruffle-ended ameloblasts, which is caused by the release of hydrogen ions during enamel mineralization, is absent when *Wdr72* is ablated.

### Interactome analysis of mouse WDR72 protein *in vitro*

WDR72 has no signal peptide and is predicted to be an intracellular protein. To better identify the intracellular processes that are dependent upon WDR72, we attempted to discover which proteins interact with WDR72. C-terminal FLAG-tagged mouse WDR72 was overexpressed in HEK293 cells, followed by immunoprecipitation with anti-FLAG antibody. Following SDS-PAGE fractionation of the immunoprecipitates, six bands were excised from the gel and submitted for protein identification by mass spectrometry. From the six bands, a total of 326 proteins were identified by mass spectrometry, including 225 matches with high confidence score (*P* < 0.05) (Table S1). Some proteins were identified in more than one band. The co-immunoprecipitating proteins that potentially interact with WDR72 clustered into several GO (gene ontology) categories and KEGG (Kyoto Encyclopedia of Genes and Genomes) pathways and shown to be involved in various cellular processes (Table1). Among the 8 KEGG pathways with more than twofold enrichment, the endocytosis (hsa04144) pathway particularly caught our attention, since the reabsorption of enamel proteins by maturation stage ameloblasts relies upon endocytosis and appears to be deficient in the *Wdr72* null mice. Eight proteins involved in endocytosis (HSPA1L, AP2B1, AP2A1, HSPA6, HSPA1A, HSPA1B, CLTC, and HSPA8) were detected, suggesting that WDR72 might be associated with the protein complex involved in clathrin-mediated endocytosis (CME) or vesicle trafficking by maturation stage ameloblasts. Although not being clustered into a pathway with significant fold enrichment, some proteins involved in ubiquitination, such as UBC (ubiquitin), UBA1 (ubiquitin-like modifier activating enzyme 1), and MIB1 (mindbomb E3 ubiquitin protein ligase 1), were identified with significant MASCOT Score (≥66), suggesting that WDR72 might be involved in ubiquitination process or associated with ubiquitinated proteins (Table1).

**Table 1:** Gene ontology analysis of WDR72 potential interacting proteins identified by affinity purification-tandem mass spectrometry

KEGG_PATHWAY terms	Genes	Fold enrichment
hsa03040:Spliceosome	EFTUD2, HSPA1A, SNW1, HSPA1B, CDC5L, DDX5, SF3A1, HNRNPU, SF3B3, SART1, PRPF6, SF3B2, HSPA1L, HNRNPM, SF3B1, AQR, PRPF8, CDC40, SNRNP200, HSPA6, ACIN1, THOC2, HSPA8	10.82752613
hsa00970:Aminoacyl-tRNA biosynthesis	IARS, RARS, LARS, EPRS, VARS, KARS, MARS	10.58744795
hsa00020:Citrate cycle (TCA cycle)	DLST, OGDHL, ACLY, DLAT, OGDH	10.00196696
hsa03018:RNA degradation	EXOSC10, EDC4, CNOT1, XRN1, XRN2, HSPA9	6.527599487
hsa00290:Valine, leucine, and isoleucine biosynthesis	IARS, LARS, VARS	16.91241685
hsa03450:Nonhomologous end-joining	XRCC6, PRKDC, RAD50	14.31050657
hsa04612:Antigen processing and presentation	HSPA1L, HSPA6, HSPA1A, HSPA5, HSPA1B, HSPA8	3.735674405
hsa04144:Endocytosis	HSPA1L, AP2B1, AP2A1, HSPA6, HSPA1A, HSPA1B, CLTC, HSPA8	2.359159597

KEGG, Kyoto Encyclopedia of Genes and Genomes.

## Discussion

Since mutations in *WDR72* (15q21.3) were first identified to cause autosomal recessive hypomaturation AI, there have been seven different disease-causing mutations identified in 10 families (El-Sayed et al. 2009, 2011; Lee et al. 2010; Wright et al. 2011; Kuechler et al. 2012; Katsura et al. 2014). All of the mutations are located sporadically throughout the gene (exons 8, 10, 12, 15, 16, and 17) and truncate the protein (either nonsense or frameshift mutations) if translated. However, the mutant transcripts all display premature stop codons prior to the last coding exon and presumably undergo nonsense-mediated decay (Bhuvanagiri et al. 2010). Also, the lack of missense mutations and the dispersed distribution of reported mutations throughout the gene suggests a disease phenotype due to a complete depletion of WDR72 protein rather than production of toxic truncated proteins. The *Wdr72* null mice we report here exhibit significant hypomaturation defects of dental enamel, with minimal ameloblast cell pathology in unerupted teeth, that phenocopy a form of human AI, and support a loss of function pathological mechanism for *WDR72*-associated AI.

More than 60% of enamel mineral deposition takes place during the maturation stage of enamel formation (Wright et al. 2011). This mineralization process depends upon two major cell-matrix interactive activities. First, the organic matrix needs to be degraded by proteases and reabsorbed by the maturation stage enamel organ (Reith and Cotty 1967; Simmer et al. 2009). Second, the regulated movement of ions (calcium, phosphate, hydrogen, and bicarbonate) into and out of the extracellular matrix is required to support ion deposition onto the sides of enamel crystallites, which significantly increases enamel hardness (Wang et al. 2014a). Two proteases, MMP20 (matrix metalloproteinase 20) and KLK4 play critical roles in extracellular matrix degradation (Lu et al. 2008). *Mmp20* (Caterina et al. 2002) and *Klk4* (Simmer et al. 2009) null mice both have defective enamel, while mutations in both alleles of *MMP20* (Kim et al. 2005) or *KLK4* (Hart et al. 2004) cause autosomal recessive AI in humans. The enamel formed in *Mmp20* or *Klk4* null mice is soft and retains enamel proteins (Yamakoshi et al. 2011), demonstrating that protease activity is required for complete removal of the organic extracellular matrix. However, it seems that more protein is retained in the *Wdr72* null than in the enamel formed by *Mmp20* and *Klk4* null mice. Also, much of the residual amelogenin in the *Mmp20* and *Klk4* null enamel is relatively intact, whereas almost all of the proteins retained in the *Wdr72*^−/−^ enamel are degraded to some extent, which suggests that defective enamel maturation in *Wdr72* null mice is not due to impaired protein degradation. Therefore, the most plausible explanation for the retention of enamel proteins in *Wdr72* null mice is a failure in the mechanism by which degraded enamel proteins are reabsorbed by ameloblasts. This conclusion, however, is difficult to reconcile with the results of ultrastructural analysis of the deciduous tooth enamel from an AI patient with a biallelic *WDR72* p.S783* mutation (El-Sayed et al. 2011). Energy-dispersive X-ray analysis spectra showed normal carbon and nitrogen peaks, excluding retention of enamel matrix proteins. This is inconsistent with our findings in *Wdr72* null mice, which demonstrate a major failure in the removal of enamel proteins both histologically and by SDS-PAGE/western blot analyses. Perhaps the residual enamel proteins were lost in the human primary tooth during its years in the oral cavity prior to exfoliation.

It has been demonstrated that there are active endocytotic activities in ameloblasts, particularly at maturation stage (Sasaki 1984; Nanci et al. 1996). Therefore, it has been long thought that ameloblasts remove degraded matrix proteins through endocytosis. CME is the uptake of materials into the cell from the surface using clathrin-coated vesicles and is the best studied endocytotic mechanism in cell biology (Kirchhausen et al. 2014). WDR72 protein has no predicted signal peptide and is considered to be an intracellular protein. Also, its closest human homolog, WDR7 (Rabconnectin-3*β*), has been shown to play a role in Ca^2+^-dependent exocytosis of neurotransmitter release at synaptic vesicles (Kawabe et al. 2003). Along with the finding that *WDR72* mutations cause human hypomaturation AI, it has therefore been speculated that WDR72 might have a similar cellular function to that of WDR7 and be involved in vesicle trafficking and membrane turnover at ameloblasts, especially in endocytosis. In this study, by using affinity purification combined with mass spectrometry (AP-MS), we demonstrated that WDR72 is associated with clathrin, AP2A1, and AP2B1, the protein components mediating CME. Although it is not clear whether WDR72 directly interacts with these proteins, this association highly suggests that WDR72 is involved in endocytosis by maturation stage ameloblasts. This finding also suggests that the excess residual proteins found in the enamel of *Wdr72*^−/−^ mice are caused by impaired ameloblastic endocytosis for protein removal and explains the hypomaturation defects of dental enamel in null mice and human patients. Furthermore, we demonstrate that the connection between maturation stage ameloblasts and enamel matrices is weakened in *Wdr72*^−/−^ incisors. This disrupted cell-matrix attachment might also partly contribute to the impaired matrix removal.

In addition to the protein components of CME, there are several ubiquitination-related proteins identified in our WDR72 AP-MS results, such as UBC (ubiquitin), UBA1 (ubiquitin-like modifier activating enzyme 1), and MIB1 (mindbomb E3 ubiquitin protein ligase 1). It has been demonstrated that ubiquitination is involved in and required for specific steps of endocytosis, especially in internalization of cell surface receptors. Interestingly, it was recently discovered that WD40 repeat propellers define an ubiquitin-binding domain (UBD) (Pashkova et al. 2010). A functionally diverse set of WD40 repeat-containing proteins was shown to bind ubiquitin in a similar fashion (Villamil et al. 2013). Therefore, it is possible that WDR72, which contains a *β*-propeller structure, interacts with ubiquitin and contributes to ubiquitination-dependent endocytosis. However, further investigation of WDR72 cellular functions at the molecular level needs to be conducted to test this hypothesis.

Specific ion transporters and related molecules, such as SLC24A4 (solute carrier family 24, member 4), STIM1 (stromal interaction molecule 1), and ORAI1 (ORAI calcium release-activated calcium modulator 1) are involved in ion transport during enamel maturation. Mutations in *SLC24A4*, *STIM1*, and *ORAI1* cause human hypomaturation AI, presumably due to impeded ion movement (Feske 2009; Parry et al. 2013). Our finding that the subcellular localization of NCKX4 (SLC24A4) was altered in *Wdr72*^*−/−*^ ameloblasts suggested that the hypomaturation enamel defects of *Wdr72*^*−/−*^ mice might result from the disturbance of calcium excretion from ameloblasts. However, the ectopic mineralized deposits covering the *Wdr72*^*−/−*^ enamel surface indicated that calcium transport was not completely impeded, but perhaps misdirected. Retention of the protein matrix may have interfered with the penetration of ions into the enamel layer or simply occupied the space necessary for crystal growth. The ameloblasts’ ion transport system may also have malfunctioned, due to problems with cell attachment and mislocalization of NCKX4 away from the distal membrane of the ameloblasts. Alternatively, the different patterns of NCKX4 localization in ameloblasts between wild-type and *Wdr72*^*−/−*^ mice might reflect a change of the proportion of ruffle-ended and smooth-ended ameloblasts during modulation, presumably leading to an altered NCKX4 immunoreactivity detected at cytoplasm rather than distal membranes of ameloblasts. It has been demonstrated in rat incisors that, during maturation stage, 50% of ameloblasts are ruffle-ended, 25% smooth-ended, and 25% transition from smooth-ended to ruffle-ended (Smith 1998). Also, the ruffle-ended ameloblasts are thought to be responsible for protein removal. Therefore, it is possible that depletion of WDR72 may cause an increased proportion of smooth-ended ameloblasts with a reduction of ruffle-ended ameloblasts, which leads to insufficient protein removal and hypomaturation defects. This hypothesis was further supported by the finding that the *Wdr72*^*−/−*^ incisor lost the normal banding pattern of Methyl Red staining, presumably due to a potential failure in ameloblastic modulation or a dominance of smooth-ended ameloblasts throughout the maturation stage. However, further investigation of ameloblastic modulation during enamel maturation of *Wdr72*^*−/−*^ mice needs to be conducted to test this hypothesis of disease mechanism.

The prominent cytoplasmic localization of SLC24A4, especially at the supranuclear endosome area in *Wdr72* null ameloblasts, suggested that the regulation of membrane protein trafficking might be impaired when WDR72 was ablated. Interestingly, mutations in WDR7, the closest homolog of WDR72, have been demonstrated to cause disrupted endocytic trafficking and accumulation of membrane proteins in late endosomes in Drosophila (Yan et al. 2009) and mammalian (Sethi et al. 2010) cells. WDR72 might have a similar function to that of WDR7 and regulate vesicle trafficking and membrane turnover in ameloblastic endocytosis, which is consistent with our *in vitro* finding that WDR72 protein is associated with endocytosis-related proteins.

Almost all the genes encoding enamel matrix proteins and proteinases exhibit a tooth-specific expression pattern. *Enam* (enamelin), *Mmp20* (matrix metalloproteinase 20), and *Klk4* (kallikrein-related peptidase 4) show only trace expression in nondental tissues (Begue-Kirn et al. 1998; Hu et al. 2008; Simmer et al. 2011). Mutations in these genes cause nonsyndromic (isolated) AI without clinically detectable abnormalities of other organ systems in human patients (Chan et al. 2011). Also, the specific gene knockout mice show no systemic anomalies (only dental enamel defects), and these genes have been found to be pseudogenized in vertebrates that have lost the ability to make teeth during evolution (Meredith et al. 2009, 2011, 2013; Kawasaki et al. 2014). However, *WDR72* is also expressed in the kidney. The human expressed sequence tag (EST) database lists 46 *WDR72* ESTs (out of 3,328,811) for normal tissues. It also lists 31 ESTs out of 210,778 (147 *per* million) from kidney. Nevertheless, in spite of its expression in kidney, human mutations in both *WDR72* alleles cause isolated enamel defects without reported involvement of other organ systems. A recent mutational analysis reported a homozygous *WDR72* nonsense mutation (p.K330*) in two siblings with hypomaturation AI, mild short stature, and speech delay. However, the consanguinity of the family as well as the lack of segregation evidence makes it likely that the nondental phenotypes are independent traits from the enamel defects, and may not be caused by the *WDR72* mutation. Consistent with the human cases, our *Wdr72* null mice are viable and fertile without apparent abnormalities of major organ systems, except defective dental enamel, which further demonstrates that WDR72 might be unimportant for the development and physiological functioning of nondental tissues. It is possible that there might be compensatory mechanisms due to gene redundancy in these tissues or that the developmental process of dental enamel formation is particularly sensitive to the lack of WDR72. Interestingly, several genetic association studies have shown that *WDR72* is one of many loci associated with kidney function and chronic kidney diseases (Kottgen et al. 2010; Franceschini et al. 2014). WDR72 is well conserved in birds, such as chickens and finches, suggesting that there are selective pressures that maintain WDR72 in birds, which have evolutionarily lost their teeth. Therefore, why the loss of WDR72 only causes enamel defects remains puzzling, and further investigations of WDR72 in nondental tissues are required to answer this question.

## Supporting Information

**Figure S1.** *Wdr72* knockout/NLS-*lac*Z knockin DNA and protein sequences. **Figure S2.** Hemimandibles and molars of D14 *Wdr72* mice. **Figure S3.** Whole-mount X-gal histostaining of 5-week-old *Wdr72*^*+/−*^ mice. **Figure S4.** *β*-gal immunostaining of ameloblasts in Day 14 *Wdr72*^*−/−*^ mandibular incisors. **Figure S5.** bSEMs of 7-week mouse mandibular incisors. **Figure S6.** bSEMs of 7-week mandibular incisor cross-sections at 1 mm increments. **Figure S7.** Knoop hardness testing of 7-week mandibular incisors. **Figure S8.** Histology of D5 maxillary molars. **Figure S9.** Histology of D11 maxillary molars. **Figure S10.** Histology of D14 maxillary molars. **Figure S11.** (A) Histology of 7-week *Wdr72*^*+/+*^ mandibular incisor (#1). Histology of 7-week *Wdr72*^*−/−*^ mandibular incisor (B) (#9), (C) (#10), (D) (#11), and (E) (#8). **Figure S12.** Immunohistochemistry of NCKX4 on wild-type D11 maxillary molars. **Figure S13.** Immunohistochemistry of NCKX4 on *Wdr72*^*−/−*^ D11 maxillary molars. **Figure S14.** Immunohistochemistry of NCKX4 on D14 mandibular incisors. **Table S1.** Mass spectrometry identifications of proteins pulled down with FLAG-tagged recombinant mouse WDR72 expressed in HEK293 cells and immunoprecipitated with anti-FLAG antibody.
