# Supplementary material for: Critical roles for WDR72 in calcium transport and matrix protein removal during enamel maturation
Source: Mol Genet Genomic Med. 2015 Mar 29;3(4):302–19. doi: 10.1002/mgg3.143 (PMC4521966; doi:10.1002/mgg3.143)
Supplement: Supplementary file 1 [file mgg30003-0302-sd1.pdf]

# Supplemental Data

**Figure S1.** *Wdr72* knockout/NLS-*lac*Z knockin DNA and protein sequences.

**Figure S2.** Hemi-mandibles and molars of D14 *Wdr72* mice.

**Figure S3.** Whole mount X-gal histostaining of 5-week-old *Wdr72*$^{+/-}$ mice.

**Figure S4.** β-gal immunostaining of ameloblasts in Day 14 *Wdr72*$^{-/-}$ mandibular incisors.

**Figure S5.** bSEMs of 7-week mouse mandibular incisors.

**Figure S6.** bSEMs of 7-week mandibular incisor cross-sections at 1 mm increments.

**Figure S7.** Knoop hardness testing of 7-week mandibular incisors.

**Figure S8.** Histology of D5 maxillary molars.

**Figure S9.** Histology of D11 maxillary molars.

**Figure S10.** Histology of D14 maxillary molars.

**Figure S11a.** Histology of 7-week *Wdr72*$^{+/+}$ mandibular incisor (#1).

**Figure S11b.** Histology of 7-week *Wdr72*$^{-/-}$ mandibular incisor (#9).

**Figure S11c.** Histology of 7-week *Wdr72*$^{-/-}$ mandibular incisor (#10).

**Figure S11d.** Histology of 7-week *Wdr72*$^{-/-}$ mandibular incisor (#11).

**Figure S11e.** Histology of 7-week *Wdr72*$^{-/-}$ mandibular incisor (#8).

**Figure S12.** Immunohistochemistry of NCKX4 on wild-type D11 maxillary molars.

**Figure S13.** Immunohistochemistry of NCKX4 on *Wdr72*$^{-/-}$ D11 maxillary molars.

**Figure S14.** Immunohistochemistry of NCKX4 on D14 mandibular incisors.

**Table S1.** Mass spectrometry identifications of proteins pulled down with FLAG-tagged recombinant mouse WDR72 expressed in HEK293 cells and immunoprecipitated with anti-FLAG antibody.

## Fig. S1a: *Wdr72* sequence that was deleted

ATGAGGGGTGCTCTGCAGGCTGTGGCCCTCTGGGGAAGAAAAGCTCCTCCCCACAGCATCACGGCCATCATGA
TCACAGATGACCAGCAAACCATAGTGACTGGAAGTCAGGAGGGTCAGCTCTGCCTCTGGAGCCTCTCACCTGA
ACTGAAGGTAAGTAAAGTCGCAGGGATTAAGTGTGTCCACTCTGCTTCTCAGACCTACAGTAATGGTAGAGGC
CTTGTGTGAAGGTGGGAAAGGTGTTCATTATAGAACAAATCAAGATGTTATGAGAAAATTATGAATAGCGTTA
ATAATTTCTGCTCCCAGCTAAGTATTATCACCCTTCGAGCTGCACTTACTTTTACTATTCTCTATTCTGAATT
ATATGTGTGAGTAAGAGCTATAGACGTTTAAGTATAGTTCTAAAGAATAATGGCCCATTAAAATAAACAAAAG
GCACTCAGAACAAATCAAGATGTTA

## Fig. S1b: Sequence that replaced deleted *Wdr72* sequence

**ATG**GACCCTGTTGTGCTGCAAAGGAGAGACTGGGAGAACCCTGGAGTGACCCAGCTCAACAGACTGGCTGCCC
ACCCTCCCTTTGCCTCTTGGAGGAACTCTGAGGAAGCCAGGACAGACAGGCCCAGCCAGCAGCTCAGGTCTCT
CAATGGAGAGTGGAGGTTTGCCTGGTTCCCTGCCCCTGAAGCTGTGCCTGAGTCTTGGCTGGAGTGTGACCTC
CCAGAGGCAGTTCCCAAGAAGAAGAGGAAAGTTGAGGCTGACACTGTTGTGGTGCCAAGCAACTGGCAGATGC
ATGGCTATGATGCCCCCATCTACACCAATGTCACCTACCCCATCACTGTGAACCCCCCTTTTGTGCCCACTGA
GAACCCCACTGGCTGCTACAGCCTGACCTTCAATGTTGATGAGAGCTGGCTGCAAGAAGGCCAGACCAGGATC
ATCTTTGATGGAGTCAACTCTGCCTTCCACCTCTGGTGCAATGGCAGGTGGGTTGGCTATGGCCAAGACAGCA
GGCTGCCCTCTGAGTTTGACCTCTCTGCCTTCCTCAGAGCTGGAGAGAACAGGCTGGCTGTCATGGTGCTCAG
GTGGTCTGATGGCAGCTACCTGGAAGACCAAGACATGTGGAGGATGTCTGGCATCTTCAGGGATGTGAGCCTG
CTGCACAAGCCCACCACCCAGATTTCTGACTTCCATGTTGCCACCAGGTTCAATGATGACTTCAGCAGAGCTG
TGCTGGAGGCTGAGGTGCAGATGTGTGGAGAACTCAGAGACTACCTGAGAGTCACAGTGAGCCTCTGGCAAGG
TGAGACCCAGGTGGCCTCTGGCACAGCCCCCTTTGGAGGAGAGATCATTGATGAGAGAGGAGGCTATGCTGAC
AGAGTCACCCTGAGGCTCAATGTGGAGAACCCCAAGCTGTGGTCTGCTGAGATCCCCAACCTCTACAGGGCTG
TTGTGGAGCTGCACACTGCTGATGGCACCCTGATTGAAGCTGAAGCCTGTGATGTTGGATTCAGAGAAGTCAG
GATTGAGAATGGCCTGCTGCTGCTCAATGGCAAGCCTCTGCTCATCAGGGGAGTCAACAGGCATGAGCACCAC
CCTCTGCATGGACAAGTGATGGATGAACAGACAATGGTGCAAGATATCCTGCTAATGAAGCAGAACAACTTCA
ATGCTGTCAGGTGCTCTCACTACCCCAACCACCCTCTCTGGTACACCCTGTGTGACAGGTATGGCCTGTATGT
TGTTGATGAAGCCAACATTGAGACACATGGCATGGTGCCCATGAACAGGCTCACAGATGACCCCAGGTGGCTG
CCTGCCATGTCTGAGAGAGTGACCAGGATGGTGCAGAGAGACAGGAACCACCCCTCTGTGATCATCTGGTCTC
TGGGCAATGAGTCTGGACATGGAGCCAACCATGATGCTCTCTACAGGTGGATCAAGTCTGTTGACCCCAGCAG
ACCTGTGCAGTATGAAGGAGGTGGAGCAGACACCACAGCCACAGACATCATCTGCCCCATGTATGCCAGGGTT
GATGAGGACCAGCCCTTCCCTGCTGTGCCCAAGTGGAGCATCAAGAAGTGGCTCTCTCTGCCTGGAGAGACCA
GACCTCTGATCCTGTGTGAATATGCACATGCAATGGGCAACTCTCTGGGAGGCTTTGCCAAGTACTGGCAAGC
CTTCAGACAGTACCCCAGGCTGCAAGGAGGATTTGTGTGGGACTGGGTGGACCAATCTCTCATCAAGTATGAT
GAGAATGGCAACCCCTGGTCTGCCTATGGAGGAGACTTTGGTGACACCCCCAATGACAGGCAGTTCTGCATGA
ATGGCCTGGTCTTTGCAGACAGGACCCCTCACCCTGCCCTCACAGAGGCCAAGCACCAGCAACAGTTCTTCCA
GTTCAGGCTGTCTGGACAGACCATTGAGGTGACATCTGAGTACCTCTTCAGGCACTCTGACAATGAGCTCCTG
CACTGGATGGTGGCCCTGGATGGCAAGCCTCTGGCTTCTGGTGAGGTGCCTCTGGATGTGGCCCCTCAAGGAA
AGCAGCTGATTGAACTGCCTGAGCTGCCTCAGCCAGAGTCTGCTGGACAACTGTGGCTAACAGTGAGGGTGGT
TCAGCCCAATGCAACAGCTTGGTCTGAGGCAGGCCACATCTCTGCATGGCAGCAGTGGAGGCTGGCTGAGAAC
CTCTCTGTGACCCTGCCTGCTGCCTCTCATGCCATCCCTCACCTGACAACATCTGAAATGGACTTCTGCATTG
AGCTGGGCAACAAGAGATGGCAGTTCAACAGGCAGTCTGGCTTCCTGTCTCAGATGTGGATTGGAGACAAGAA
GCAGCTCCTCACCCCTCTCAGGGACCAATTCACCAGGGCTCCTCTGGACAATGACATTGGAGTGTCTGAGGCC
ACCAGGATTGACCCAAATGCTTGGGTGGAGAGGTGGAAGGCTGCTGGACACTACCAGGCTGAGGCTGCCCTGC
TCCAGTGCACAGCAGACACCCTGGCTGATGCTGTTCTGATCACCACAGCCCATGCTTGGCAGCACCAAGGCAA

GACCCTGTTCATCAGCAGAAAGACCTACAGGATTGATGGCTCTGGACAGATGGCAATCACAGTGGATGTGGAG
GTTGCCTCTGACACACCTCACCCTGCAAGGATTGGCCTGAACTGTCAACTGGCACAGGTGGCTGAGAGGGTGA
ACTGGCTGGGCTTAGGCCCTCAGGAGAACTACCCTGACAGGCTGACAGCTGCCTGCTTTGACAGGTGGGACCT
GCCTCTGTCTGACATGTACACCCCTTATGTGTTCCCTTCTGAGAATGGCCTGAGGTGTGGCACCAGGGAGCTG
AACTATGGTCCTCACCAGTGGAGGGGAGACTTCCAGTTCAACATCTCCAGGTACTCTCAGCAACAGCTCATGG
AAACCTCTCACAGGCACCTGCTCCATGCAGAGGAGGGAACCTGGCTGAACATTGATGGCTTCCACATGGGCAT
TGGAGGAGATGACTCTTGGTCTCCTTCTGTGTCTGCTGAGTTCCAGTTATCTGCTGGCAGGTACCACTATCAG
CTGGTGTGGTGCCAGAAG**TAA**ACCTAATCTAGAGCTTGATGGGTGGCATCCCTGTGACCCCTCCCCAGTGCCT
CTCCTGGCCCTGGAAGTTGCCACTCCAGTGCCCACCAGCCTTGTCCT**AATAAA**ATTAAGTTGCATCATTTTGT
CTGACTAGGTGTCCTTCTATAATATTATGGGGTGGAGGGGGGTGGTATGGAGCAAGGGGCAAGTTGGGAAGAC
AACCTGTAGGGCCTGCGGGGTCTATTGGGAACCAAGCTGGAGTGCAGTGGCACAATCTTGGCTCACTGCAATC
TCCGCCTCCTGGGTTCAAGCGATTCTCCTGCCTCAGCCTCCCGAGTTGTTGGGATTCCAGGCATGCATGACCA
GGCTCAGCTAATTTTTGTTTTTTTGGTAGAGACGGGGTTTCACCATATTGGCCAGGCTGGTCTCCAACTCCTA
ATCTCAGGTGATCTACCCACCTTGGCCTCCCAAATTGCTGGGATTACAGGCGTGAACCACTGCTCCCTTCCCT
GTCCTTCTGATTTTAAAATAACTATACCAGCAGGAGGACGTCCAGACACAGCATAGGCTACCTGGCCATGCCC
AACCGGTGGGACATTTGAGTTGCTTGCTTGGCACTGTCCTCTCATGCGTTGGGTCCACTCAGTAGATGCCTGT
TGAATTGCAGATCTGCTTAAGCTCGCGAGCCTGACAGGCTCGTACGTCTCCCAGCTAAGTATTATCACCCTTC
GAGCTGCACTTACTTTTACTATTCTCTATTCTGAATTATATGTGTGAGTAAGAGCTATAGACGTTTAAGTATA
GTTCTAAAGAATAATGGCCCATTAA**AATAAA**CAAAAGGCACTCCTGAAAGCCACTATTAAC

## Fig. S1c: Deduced 1028 amino acid sequence of NLS-*lac*Z.

MDPVVLQRRDWENPGVTQLNRLAAHPPFASWRNSEEARTDRPSQQLRSLNGEWRFAWFPAPEAVPESWLECDL
PEAVPKKKRKVEADTVVVPSNWQMHGYDAPIYTNVTYPITVNPPFVPTENPTGCYSLTFNVDESWLQEGQTRI
IFDGVNSAFHLWCNGRWVGYGQDSRLPSEFDLSAFLRAGENRLAVMVLRWSDGSYLEDQDMWRMSGIFRDVSL
LHKPTTQISDFHVATRFNDDFSRAVLEAEVQMCGELRDYLRVTVSLWQGETQVASGTAPFGGEIIDERGGYAD
RVTLRLNVENPKLWSAEIPNLYRAVVELHTADGTLIEAEACDVGFREVRIENGLLLLNGKPLLIRGVNRHEHH
PLHGQVMDEQTMVQDILLMKQNNFNAVRCSHYPNHPLWYTLCDRYGLYVVDEANIETHGMVPMNRLTDDPRWL
PAMSERVTRMVQRDRNHPSVIIWSLGNESGHGANHDALYRWIKSVDPSRPVQYEGGGADTTATDIICPMYARV
DEDQPFPAVPKWSIKKWLSLPGETRPLILCEYAHAMGNSLGGFAKYWQAFRQYPRLQGGFVWDWVDQSLIKYD
ENGNPWSAYGGDFGDTPNDRQFCMNGLVFADRTPHPALTEAKHQQQFFQFRLSGQTIEVTSEYLFRHSDNELL
HWMVALDGKPLASGEVPLDVAPQGKQLIELPELPQPESAGQLWLTVRVVQPNATAWSEAGHISAWQQWRLAEN
LSVTLPAASHAIPHLTTSEMDFCIELGNKRWQFNRQSGFLSQMWIGDKKQLLTPLRDQFTRAPLDNDIGVSEA
TRIDPNAWVERWKAAGHYQAEAALLQCTADTLADAVLITTAHAWQHQGKTLFISRKTYRIDGSGQMAITVDVE
VASDTPHPARIGLNCQLAQVAERVNWLGLGPQENYPDRLTAACFDRWDLPLSDMYTPYVFPSENGLRCGTREL
NYGPHQWRGDFQFNISRYSQQQLMETSHRHLLHAEEGTWLNIDGFHMGIGGDDSWSPSVSAEFQLSAGRYHYQ
LVWCQK*

**Figure S1.** *Wdr72* knockout/NLS-*lac*Z knockin DNA and protein sequences.

***A:*** 463 basepairs of the *Wdr72* gene were deleted, including 153 coding bp (yellow) from the translation initiation codon to the end of exon 2 and 310 bp from the 5' end of intron 2.

***B:*** 3930 basepairs replaced the 463 basepairs from *Wdr72*, including 3087 of NLS-*lac*Z coding region and 843 bp of noncoding region that included 2 polyadenylation/cleavage signals (underlined).

***C:*** Deduced 1028 amino acid sequence of NLS-*lac*Z.

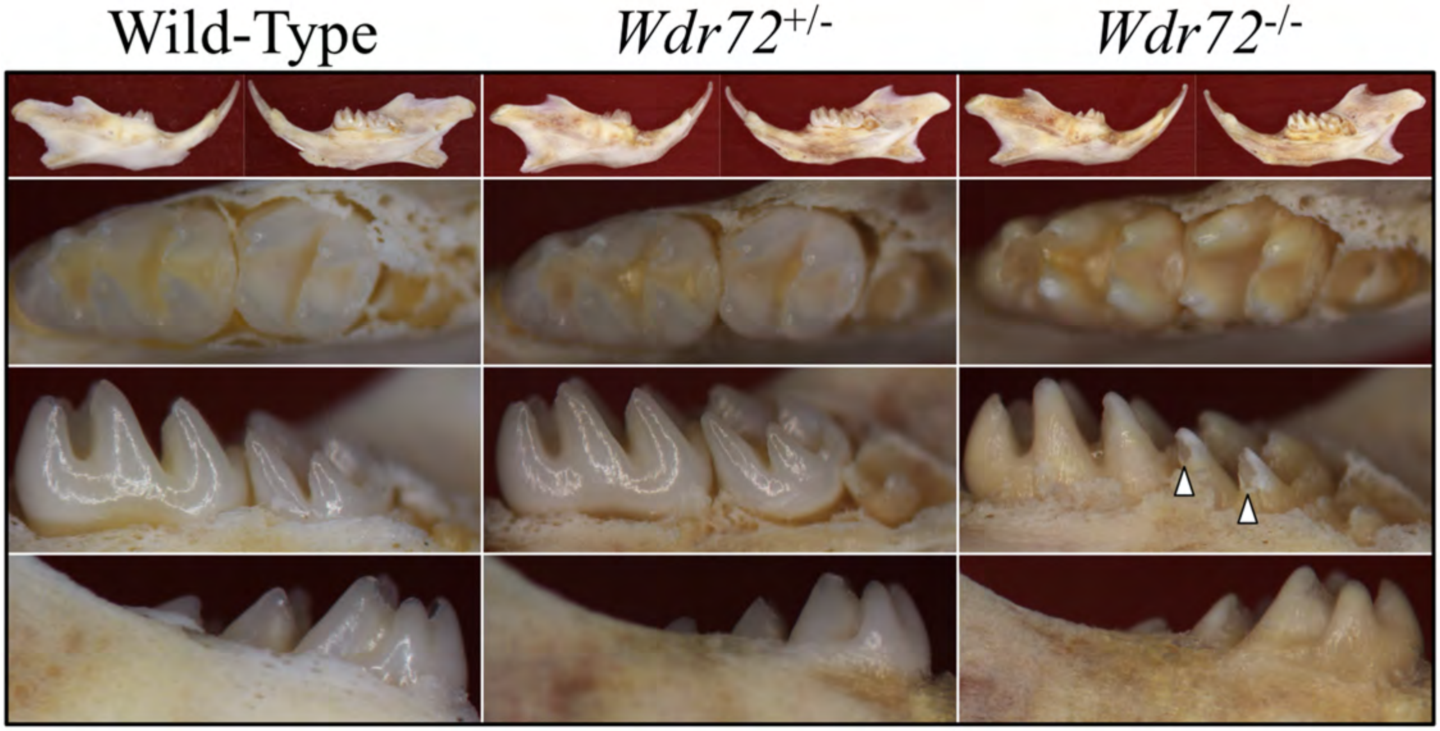


**Figure S2.** Hemi-mandibles and molars of D14 *Wdr72* mice. The *Wdr72*$^{-/-}$ molars showed rough, yellowish surfaces even prior to eruption into the oral cavity. In some areas the soft, null enamel was denuded during soft tissue removal (arrowheads).

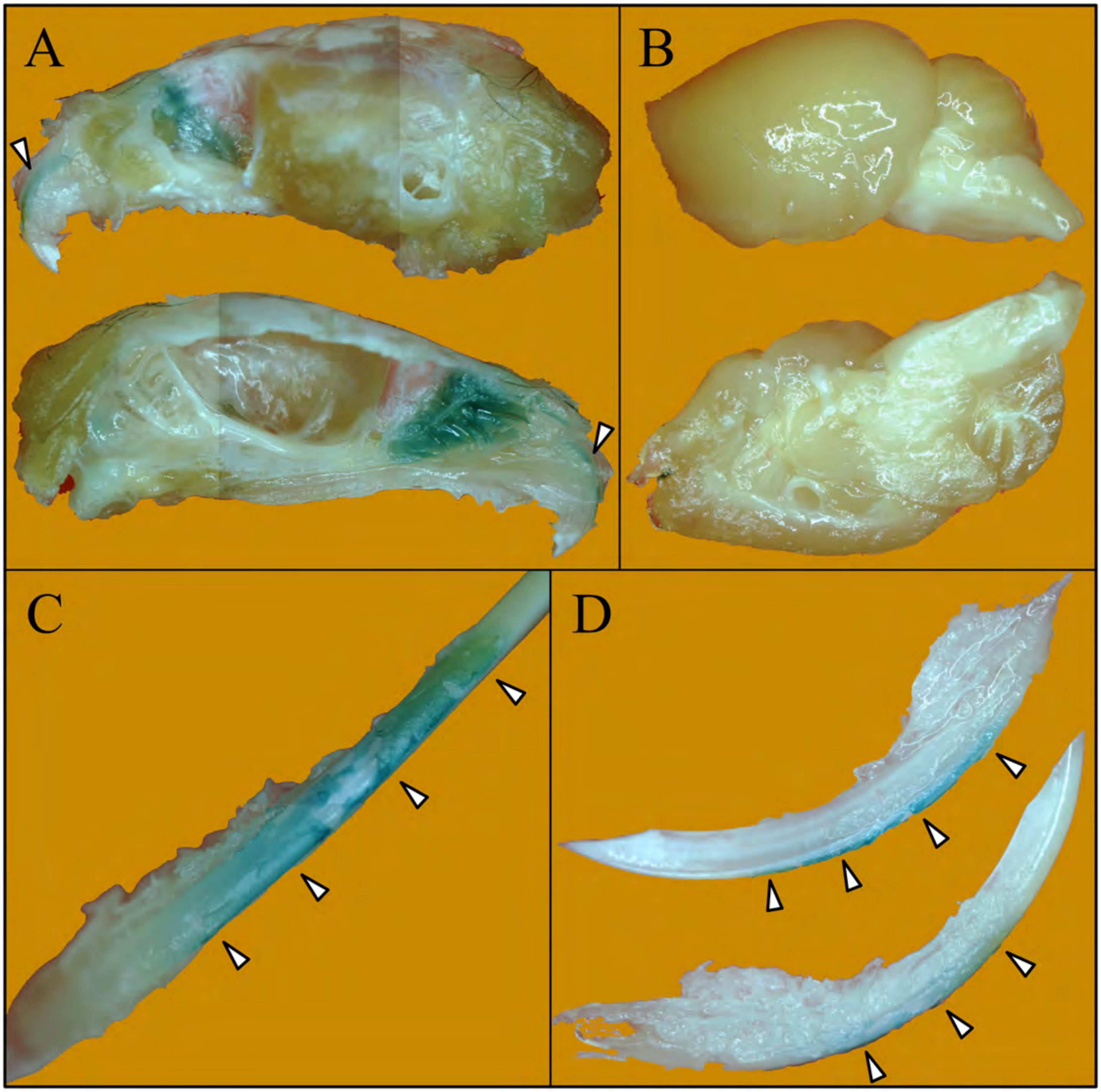


**Figure S3.** Whole mount X-gal histostaining of 5-week-old *Wdr72*$^{+/-}$ mice. ***A:*** Lateral (upper) and medial (lower) views of hemi-cranium without brain and mandible. The arrowheads indicate the positive staining (blue) of maxillary incisors. ***B:*** Lateral (upper) and medial (lower) views of a half-brain. ***C:*** Labial view of a mandibular incisor. ***D:*** Medial (upper) and lateral (lower) views of the mandibular incisor. Arrowheads indicate the positive staining is over the labial surface.

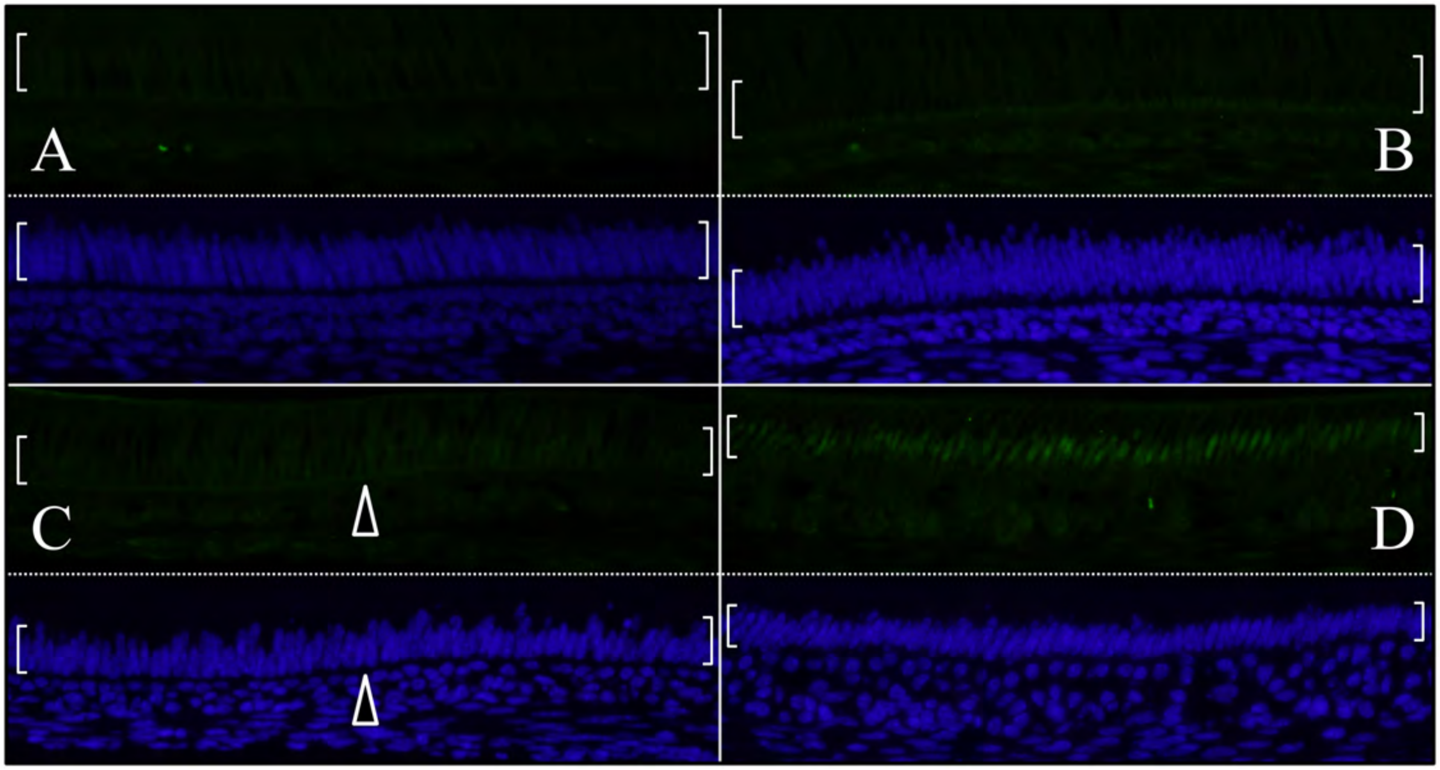


**Figure S4.** β-gal immunostaining of ameloblasts in Day 14 *Wdr72*$^{-/-}$ mandibular incisors. Four ameloblast stages are shown: early secretory (***A***), late secretory (***B***), transition (***C***), and maturation (***D***). Each upper panel shows β-gal immunoreactivity (green fluorescence in nuclei), while the lower panels show DAPI staining of nuclei (blue). The brackets indicate the palisading nuclei of ameloblasts. No signal is detected until ameloblasts hit the transition stage (***C***). Arrowheads indicate the onset of positive immunoreactivity.

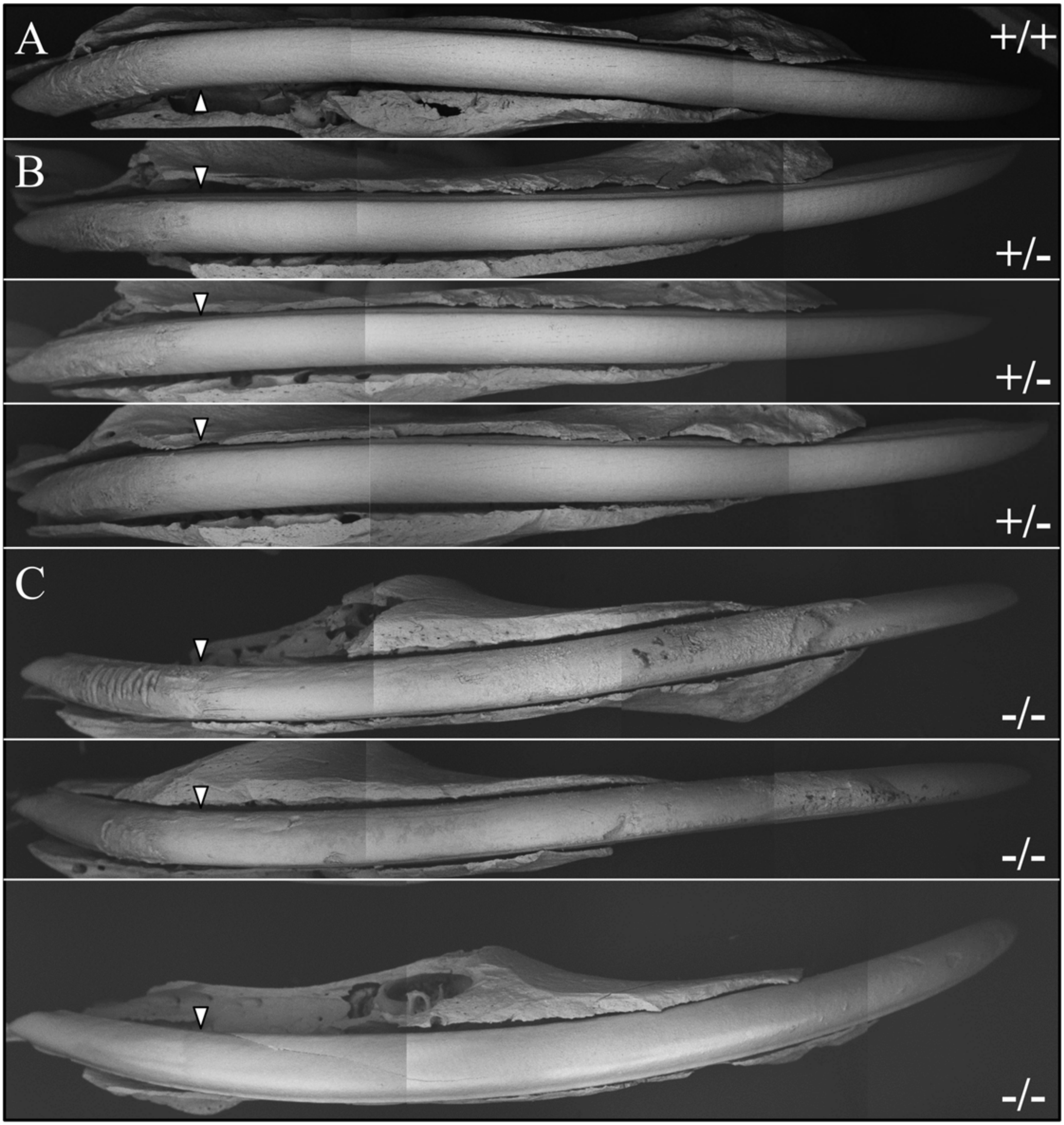


**Figure S5.** bSEMs of 7-week mouse mandibular incisors. These incisors were lightly bleached to facilitate soft tissue removal. The labial surfaces of ***A:*** wild-type (*Wdr72*$^{+/+}$); ***B:*** heterozygous (*Wdr72*$^{+/-}$); ***C:*** null (*Wdr72*$^{-/-}$) mice were examined. The apical ends of the incisors are toward the left, and the incisal ends toward the right. Bleaching was necessary because in the *Wdr72* null mice the soft tissue was partially mineralized and adherant to the enamel surface, while at the same time the underlying bulk enamel was extremely weak. Except for the bleaching, much of the null surface enamel would have stripped off during preparation, although it created some artifacts in the secretory stage enamel (left of arrowheads). While the wild-type and *Wdr7*$^{+/-}$ incisors have smooth labial enamel surfaces, the null incisors show rough surfaces with severe attrition of their incisal edges. Some of the roughness was due to damage during removal of soft tissue (scars in the surface), but most of the roughness was due to an irregular mineral crust on the enamel surface.

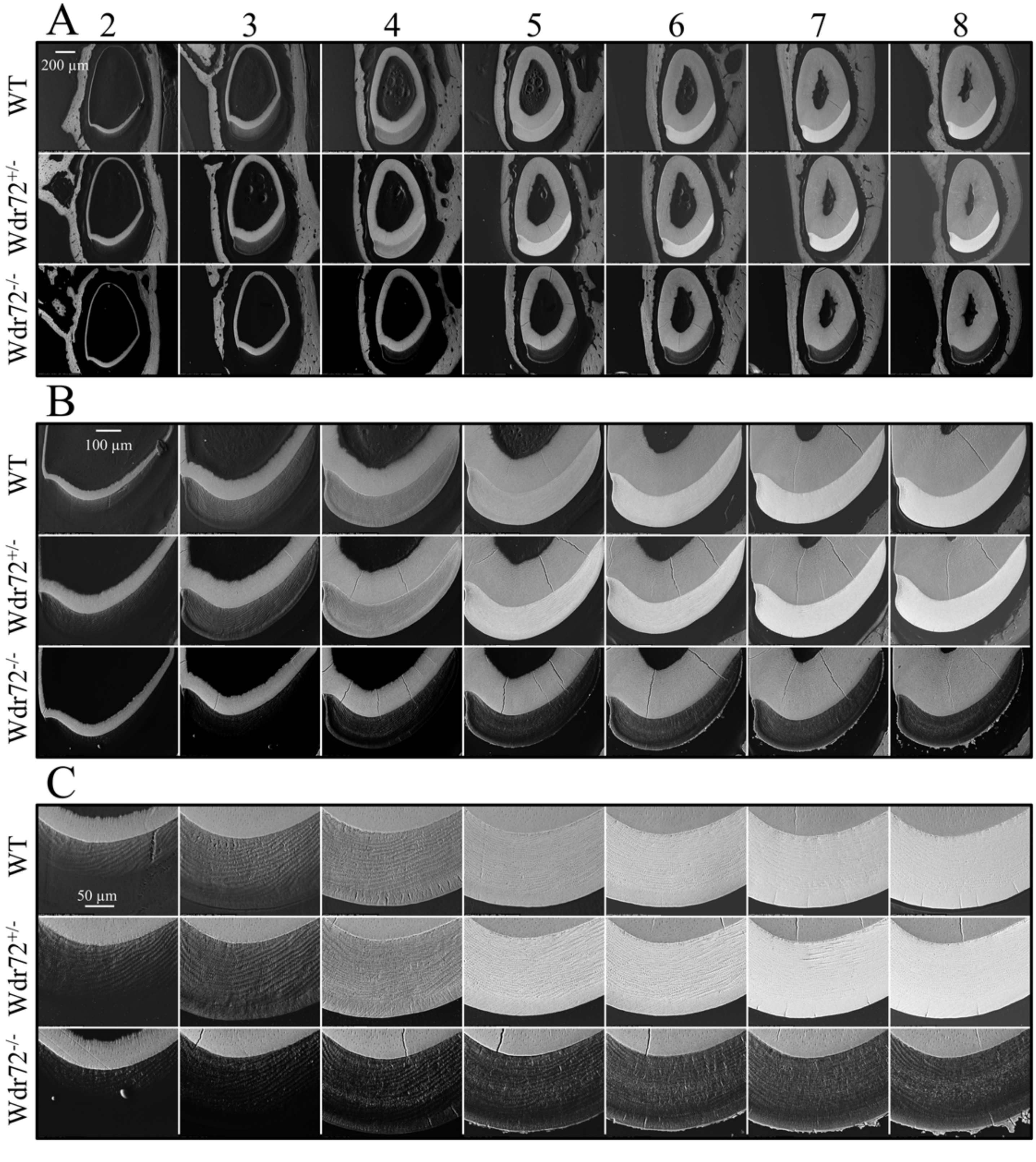


**Figure S6.** bSEMs of 7-week mandibular incisor cross-sections at 1 mm increments. Level 2 is toward the apical end of the incisor, and Level 8 the incisal end at the level of alveolar crest. The transition stage of enamel formation is located between Levels 2 and 3. While the electron density of the wild-type and *Wdr7*$^{+/-}$ enamel increased significantly at each successive level, that of *Wdr72*$^{-/-}$ enamel did not. The maturation stage enamel was significantly less electron-dense than dentin, suggesting a very severed hypomineralization defect.

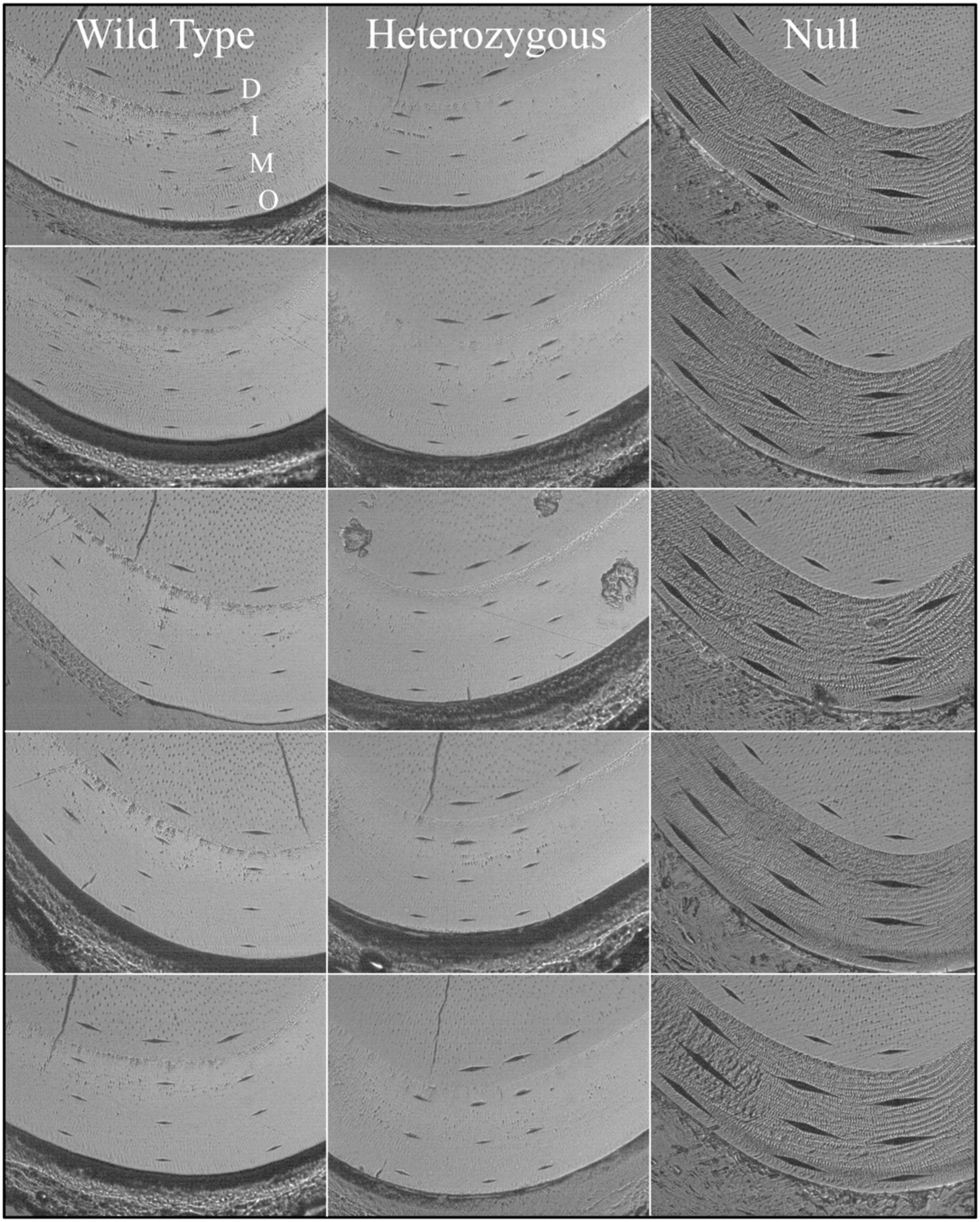


**Figure S7.** Knoop hardness testing of 7-week mandibular incisors. The complete panel of indentations on level 8 incisor cross-sections for the three genotypes are shown. The Knoop hardness values presented on Figure 6 were calculated from the measurements of these indentations. ***Key:*** D, dentin; I, inner enamel; M, middle enamel; O, outer enamel.

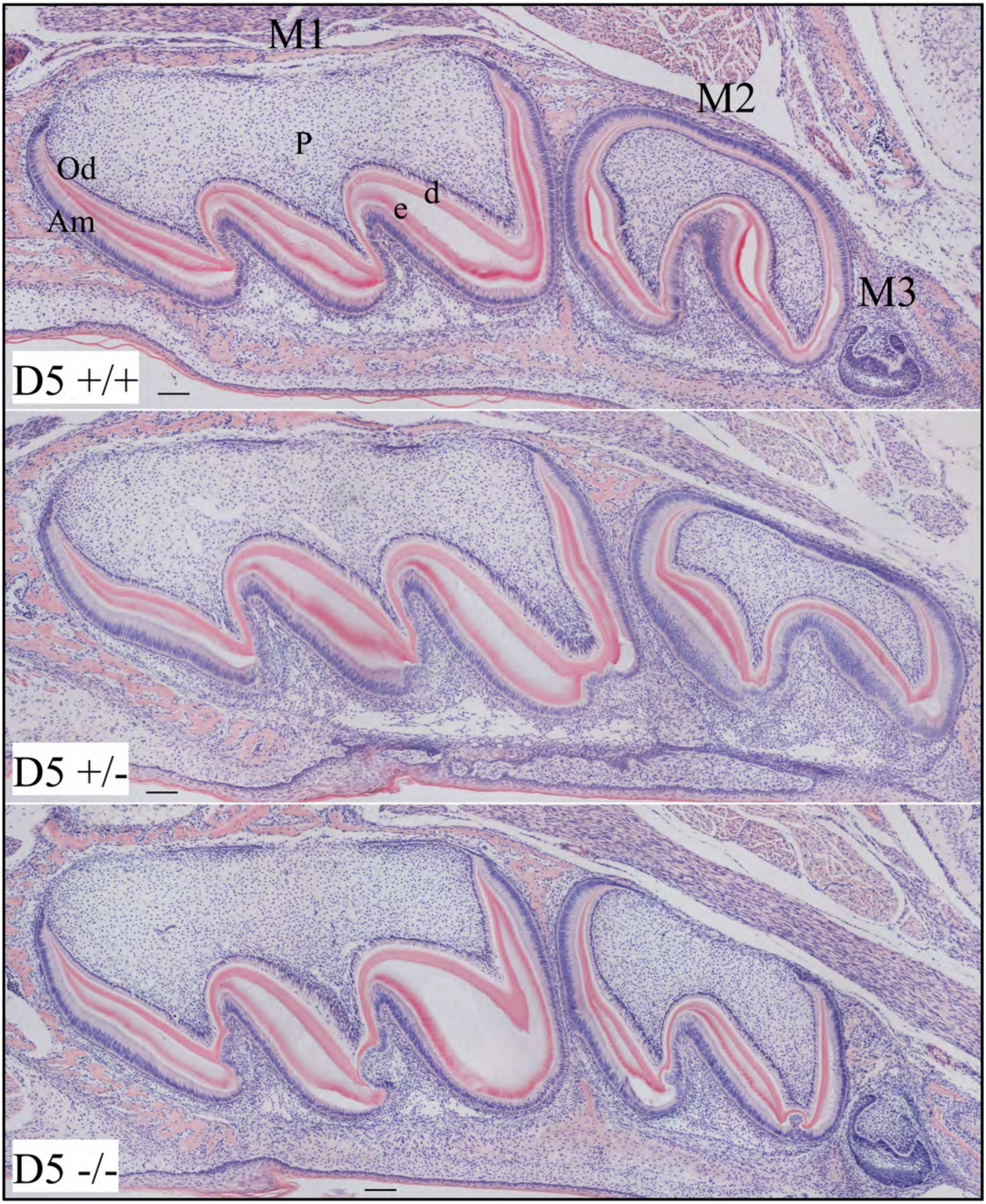


**Figure S8.** Histology of D5 maxillary molars. H&E stained sections (10×) of three genotypes (wild-type, *Wdr72*$^{+/-}$, and *Wdr72*$^{-/-}$) at D5 when first molar ameloblasts are predominantly in the secretory stage. ***Key:*** Am, ameloblasts; d, dentin; e, enamel; M1, first molar; M2, second molar; M3, third molar; Od, odontoblasts; P, dental pulp. ***Scale Bars:*** 100 μm.

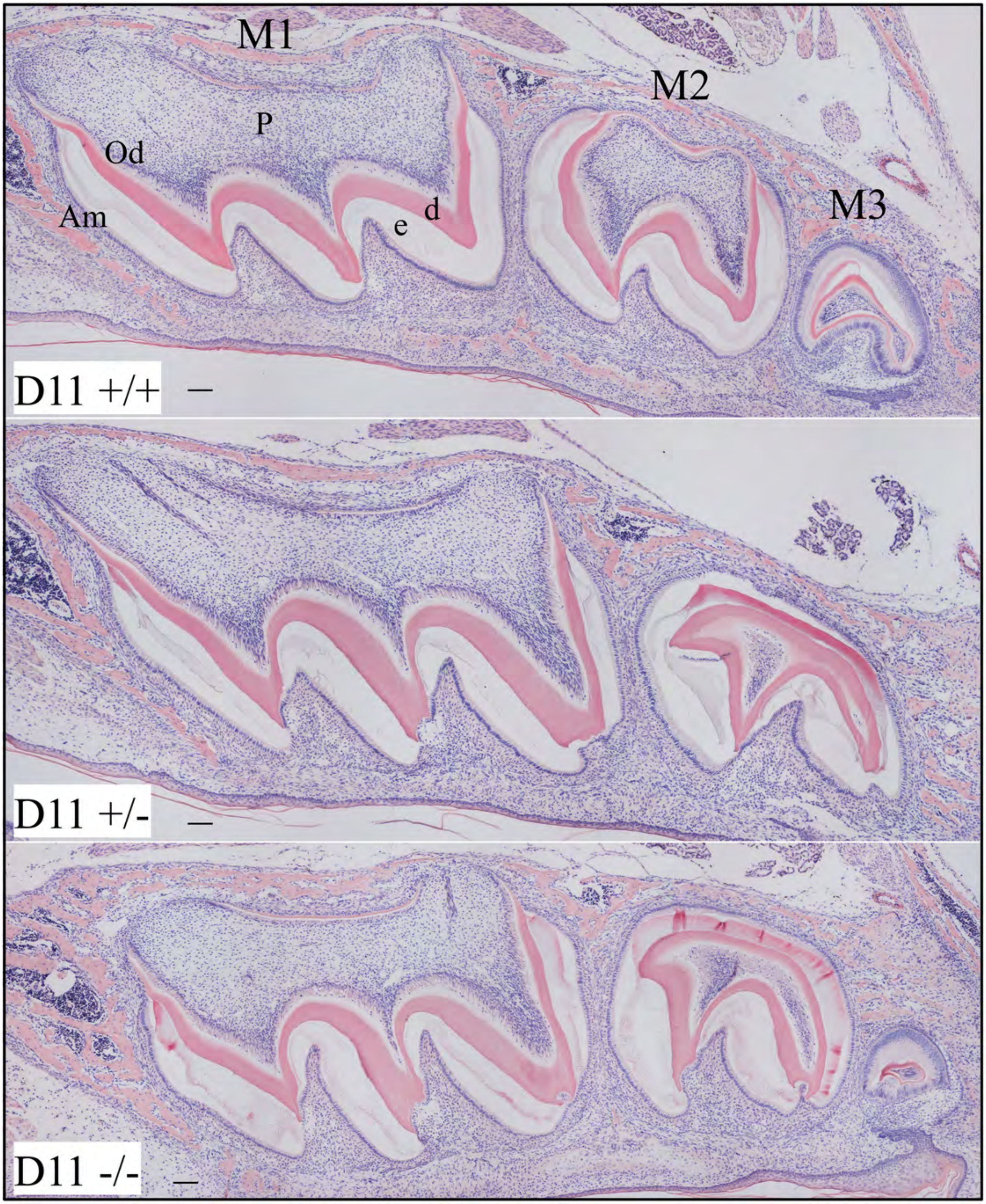


**Figure S9.** Histology of D11 maxillary molars. H&E stained sections (10×) of three genotypes (wild-type, *Wdr72*$^{+/-}$, and *Wdr72*$^{-/-}$) at D11 when first molar ameloblasts are predominantly in mid-maturation. ***Key:*** Am, ameloblasts; d, dentin; e, enamel; M1, first molar; M2, second molar; M3, third molar; Od, odontoblasts; P, dental pulp. ***Scale Bars:*** 100 µm.

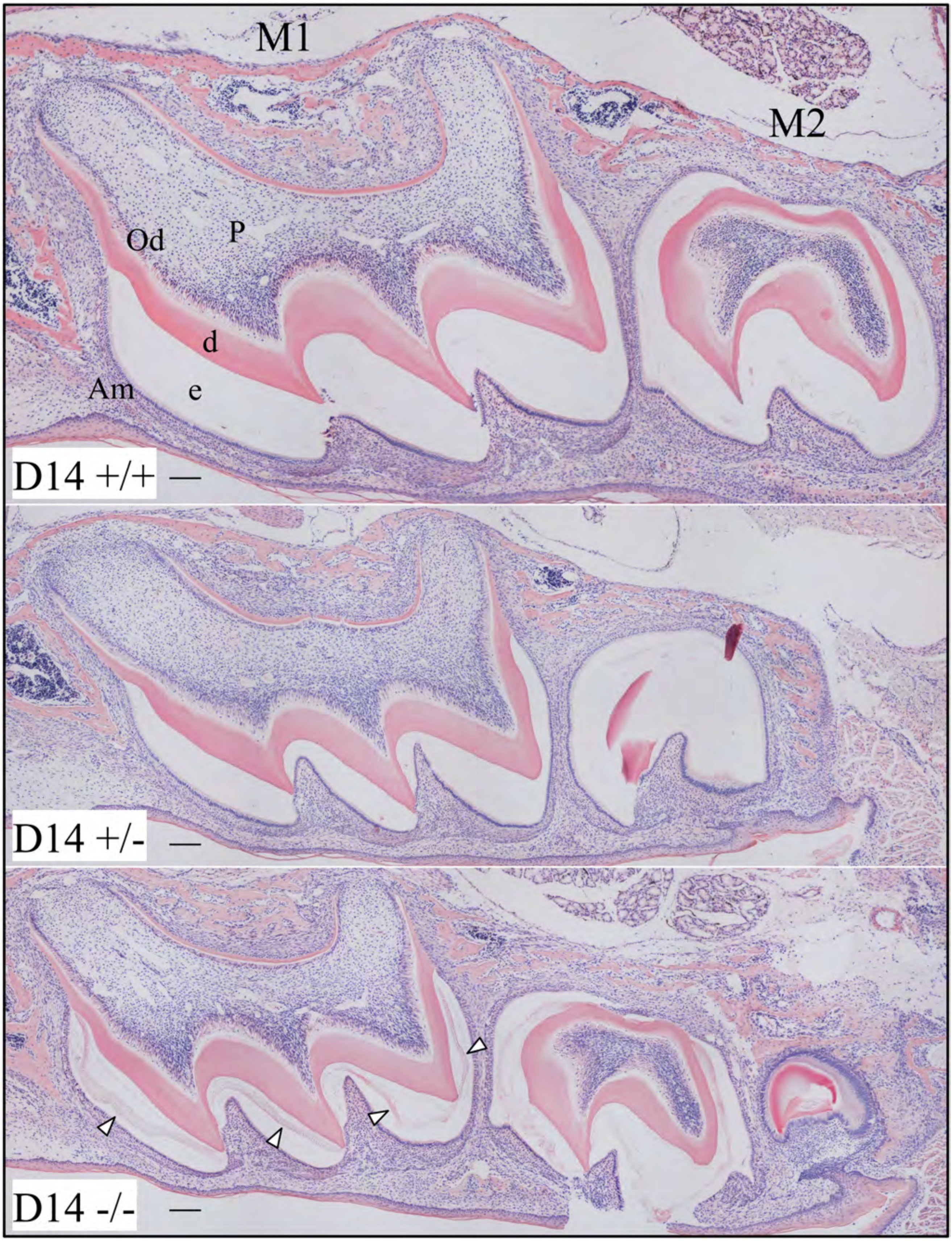


**Figure S10.** Histology of D14 maxillary molars. H&E stained sections (10×) of three genotypes (wild-type, *Wdr72*$^{+/-}$, and *Wdr72*$^{-/-}$) at D14 when first molar ameloblasts are predominantly in late maturation and about to erupt. Arrowheads indicate the presence of residual proteins in the enamel matrix even as the molar is about to erupt. ***Key:*** Am, ameloblasts; d, dentin; e, enamel; M1, first molar; M2, second molar; M3, third molar; Od, odontoblasts; P, dental pulp. ***Scale Bars:*** 100 µm.

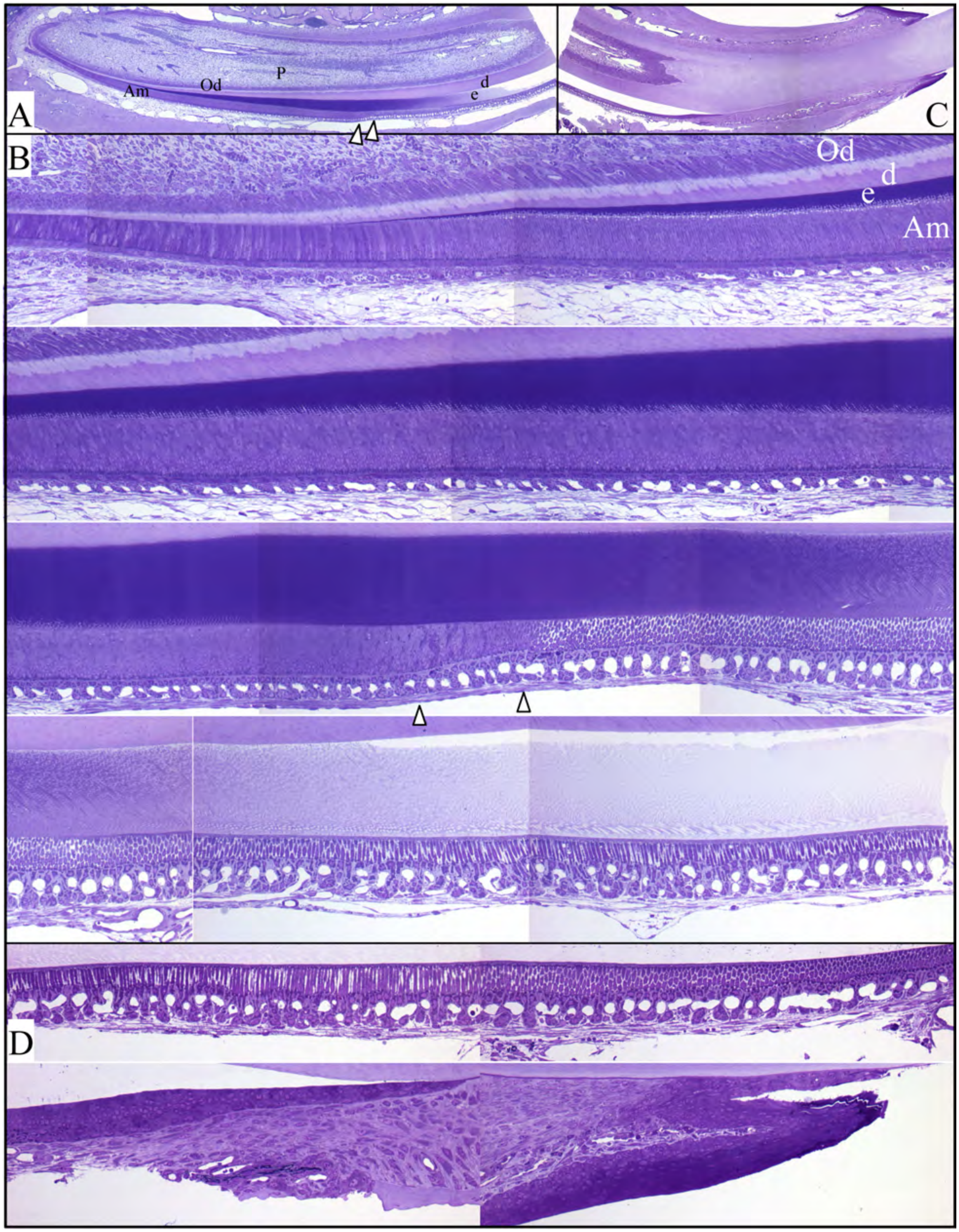


**Figure S11a.** Histology of 7-week *Wdr72*$^{+/+}$ mandibular incisor (#1). ***A:*** Low-magnification view (10×) of the basal end of the incisor. ***B:*** Higher-magnification (20×) views of the basal end of the incisor. Arrowheads mark post-secretory transition. ***C:*** Low-magnification views (10×) of the incisal end. ***D:*** Higher-magnification (20×) views of the inicisal end. All views are toluidine blue-stained longitudinal sections. The apical (cervical) end is toward the left, and the incisal end toward the right. Ameloblasts are beneath the enamel. Other panels show higher-magnification (20×) views of the incisor focusing on ameloblasts. The right end of each panel is continuous with the left end of the succeeding panel. Note that there is no artifactual separation of the enamel and the maturation stage ameloblasts. ***Key:*** Am, ameloblasts; d, dentin; e, enamel; Od, odontoblasts; P, pulp.

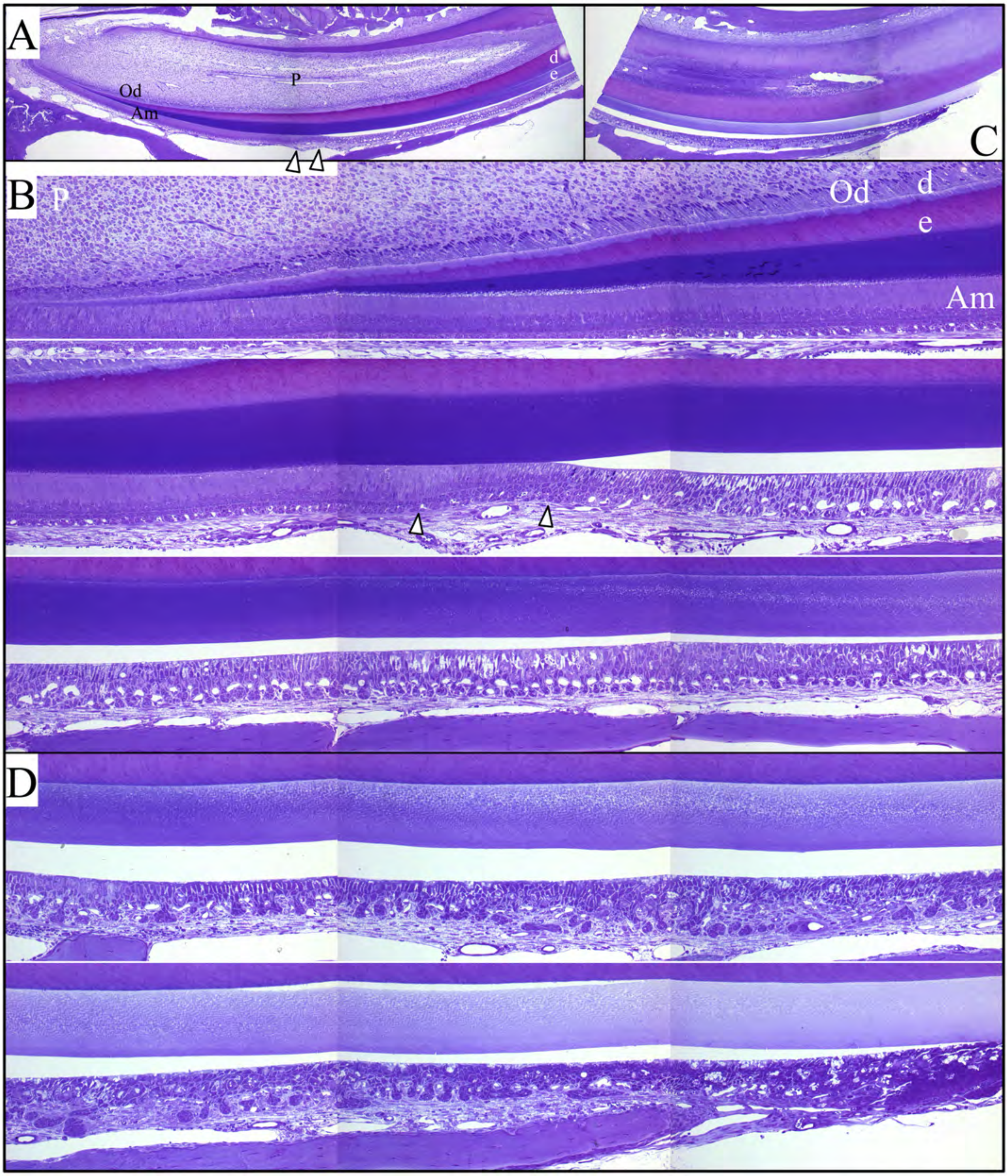


**Figure S11b.** Histology of 7-week *Wdr72*$^{-/-}$ mandibular incisor (#9). ***A:*** Low-magnification view (10×) of the basal end of the incisor. ***B:*** Higher-magnification (20×) views of the basal end of the incisor. Arrowheads mark post-secretory transition. ***C:*** Low-magnification views (10×) of the incisal end. ***D:*** Higher-magnification (20×) views of the inicisal end. All views are toluidine blue-stained longitudinal sections. Note the separation space between the maturation stage ameloblasts and the enamel. The residual enamel matrix diminishes in staining intensity only slightly during enamel maturation. Ectopic mineral is observed along the distal border of ameloblasts against the enamel space. ***Key:*** Am, ameloblasts; d, dentin; e, enamel; Od, odontoblasts; P, pulp.

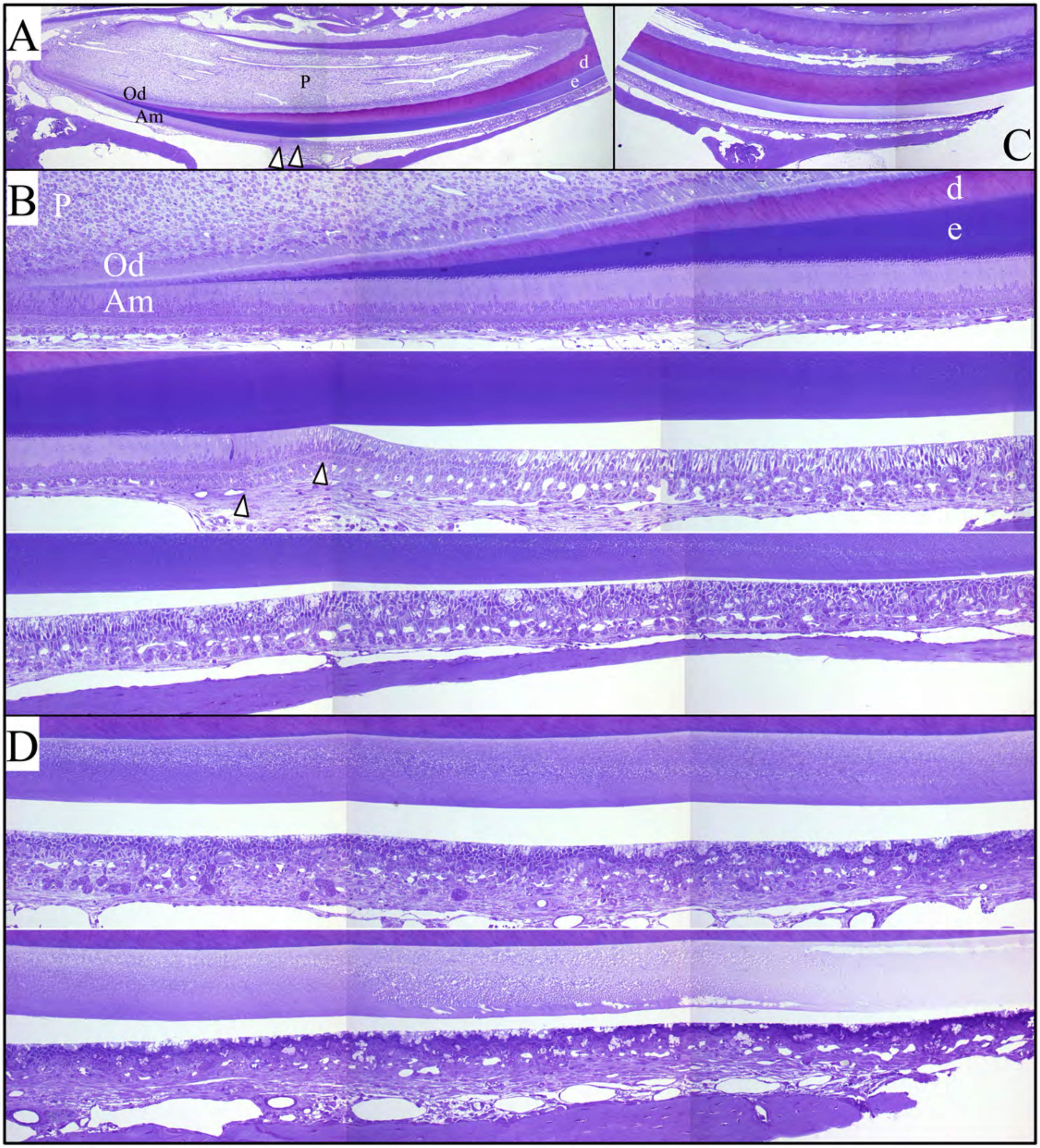


**Figure S11c.** Histology of 7-week *Wdr72*$^{-/-}$ mandibular incisor (#10). The uppermost panel shows the low-magnification views (10×) of the toluidine blue-stained longitudinal section. Arrowheads mark post-secretory transition. The apical (cervical) end is toward the left, and the incisal end the right. Ameloblasts are beneath the enamel. Other panels show higher-magnification (20×) views of the incisor focusing on ameloblasts. The right end of each panel is continuous with the left end of the succeeding panel. Although the *Wdr72*$^{-/-}$ ameloblasts showed normal morphologies without apparent cell pathology, the enamel detached from maturation stage ameloblasts after transition stage of enamel formation. Mineral projections from the enamel surface appear to interupt the layer of maturation ameloblasts (white arrows). ***Key:*** Am, ameloblasts; d, dentin; e, enamel; Od, odontoblasts; P, pulp.

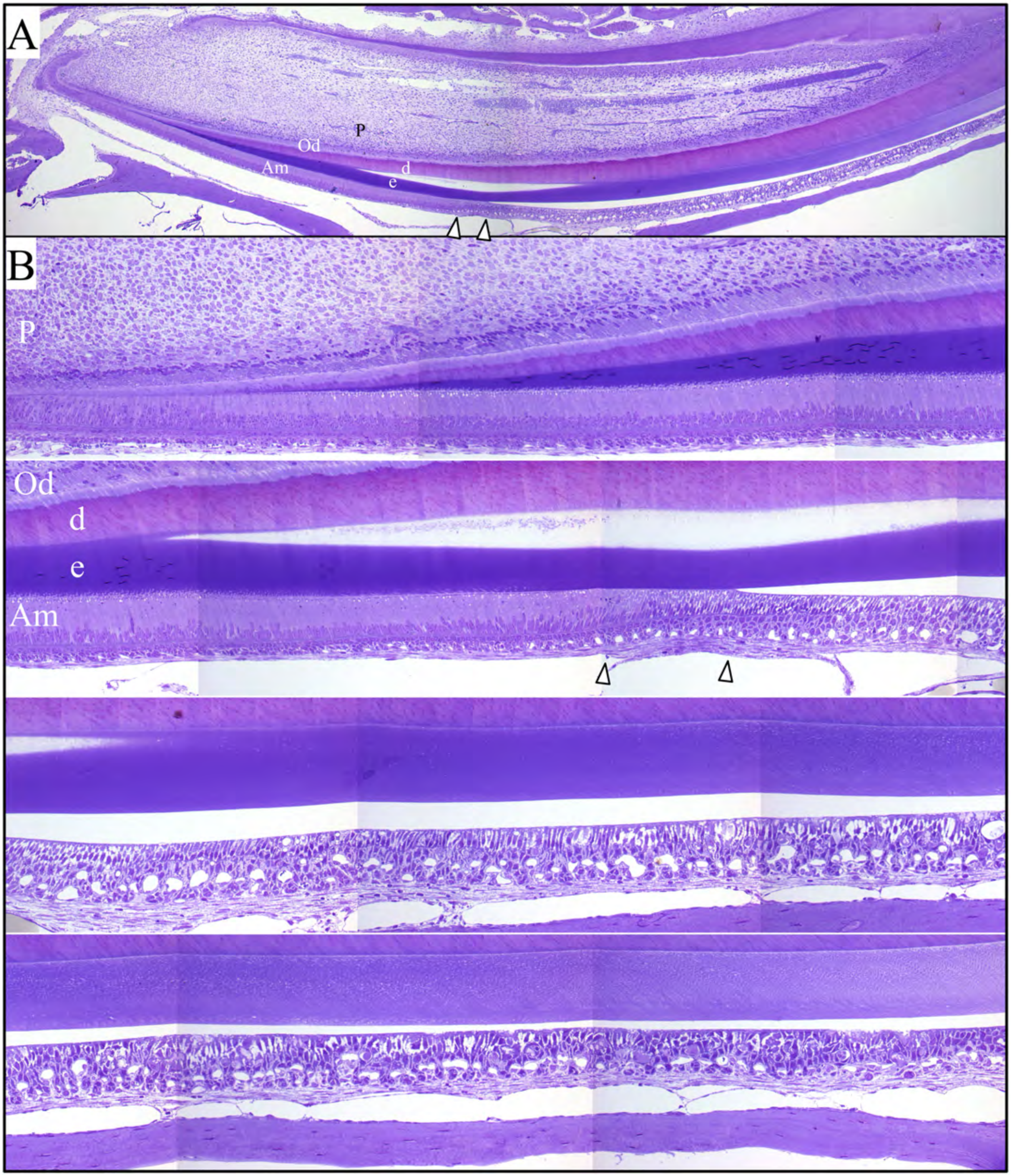


**Figure S11d.** Histology of 7-week *Wdr72*$^{-/-}$ mandibular incisor (#11). The uppermost panel shows the low-magnification views (10×) of the toluidine blue-stained longitudinal section. Arrowheads mark post-secretory transition. The apical (cervical) end is toward the left, and the incisal end the right. Ameloblasts are beneath the enamel. Other panels show higher-magnification (20×) views of the incisor focusing on ameloblasts. The right end of each panel is continuous with the left end of the succeeding panel. Although the *Wdr72*$^{-/-}$ ameloblasts showed normal morphologies without apparent cell pathology, the enamel detached from maturation stage ameloblasts after transition stage of enamel formation. Mineral projections from the enamel surface appear to interupt the layer of maturation ameloblasts (white arrows). ***Key:*** Am, ameloblasts; d, dentin; e, enamel; Od, odontoblasts; P, pulp.

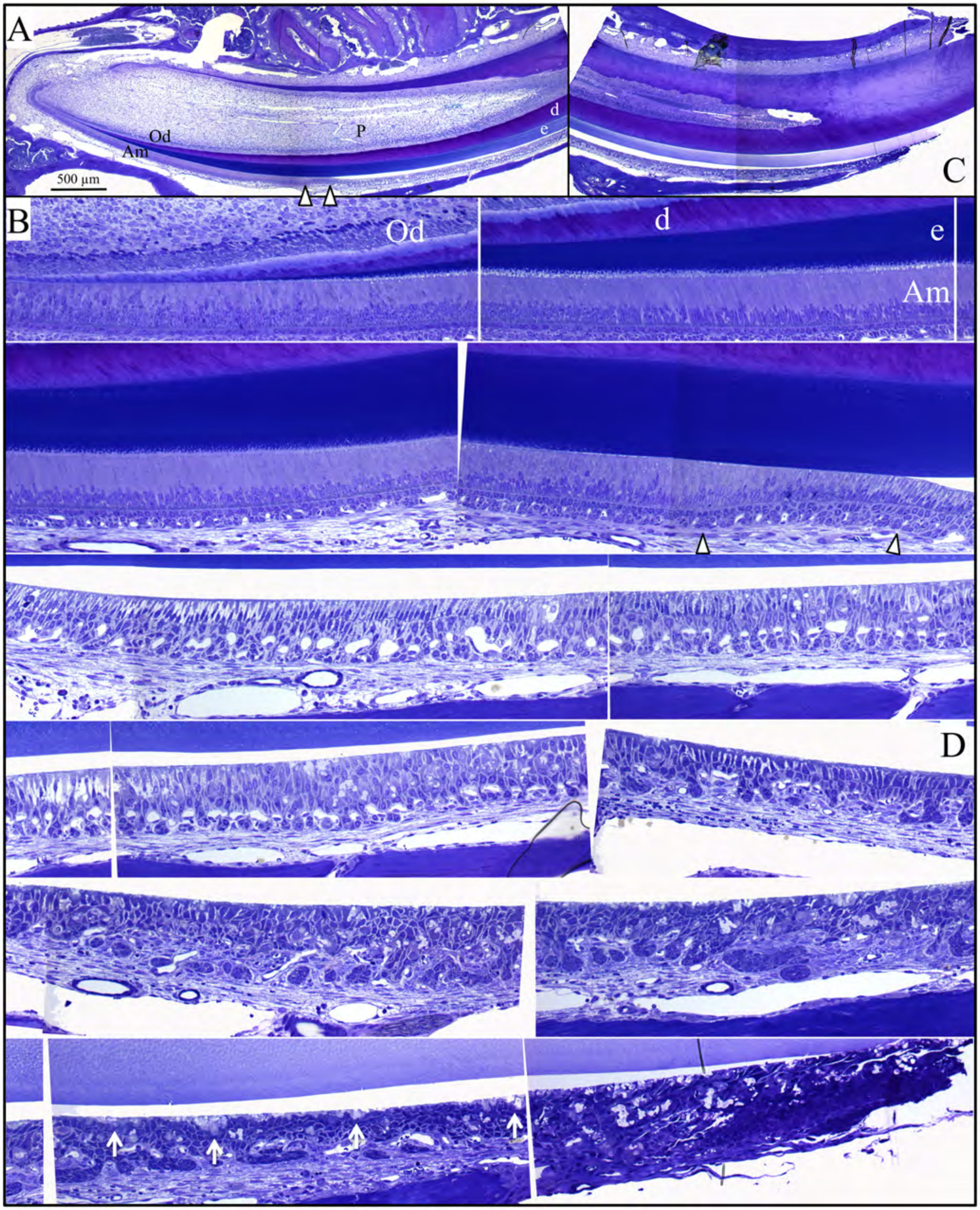


**Figure S11e.** Histology of 7-week *Wdr72*$^{-/-}$ mandibular incisor (#8). The uppermost panel shows the low-magnification views (10×) of the toluidine blue-stained longitudinal section. Arrowheads mark post-secretory transition. The apical (cervical) end is toward the left, and the incisal end the right. Ameloblasts are beneath the enamel. Other panels show higher-magnification (20×) views of the incisor focusing on ameloblasts. The right end of each panel is continuous with the left end of the succeeding panel. Although the *Wdr72*$^{-/-}$ ameloblasts showed normal morphologies without apparent cell pathology, the enamel detached from maturation stage ameloblasts after transition stage of enamel formation. Mineral projections from the enamel surface appear to interupt the layer of maturation ameloblasts (white arrows) ***Key:*** Am, ameloblasts; d, dentin; e, enamel; Od, odontoblasts; P, pulp.

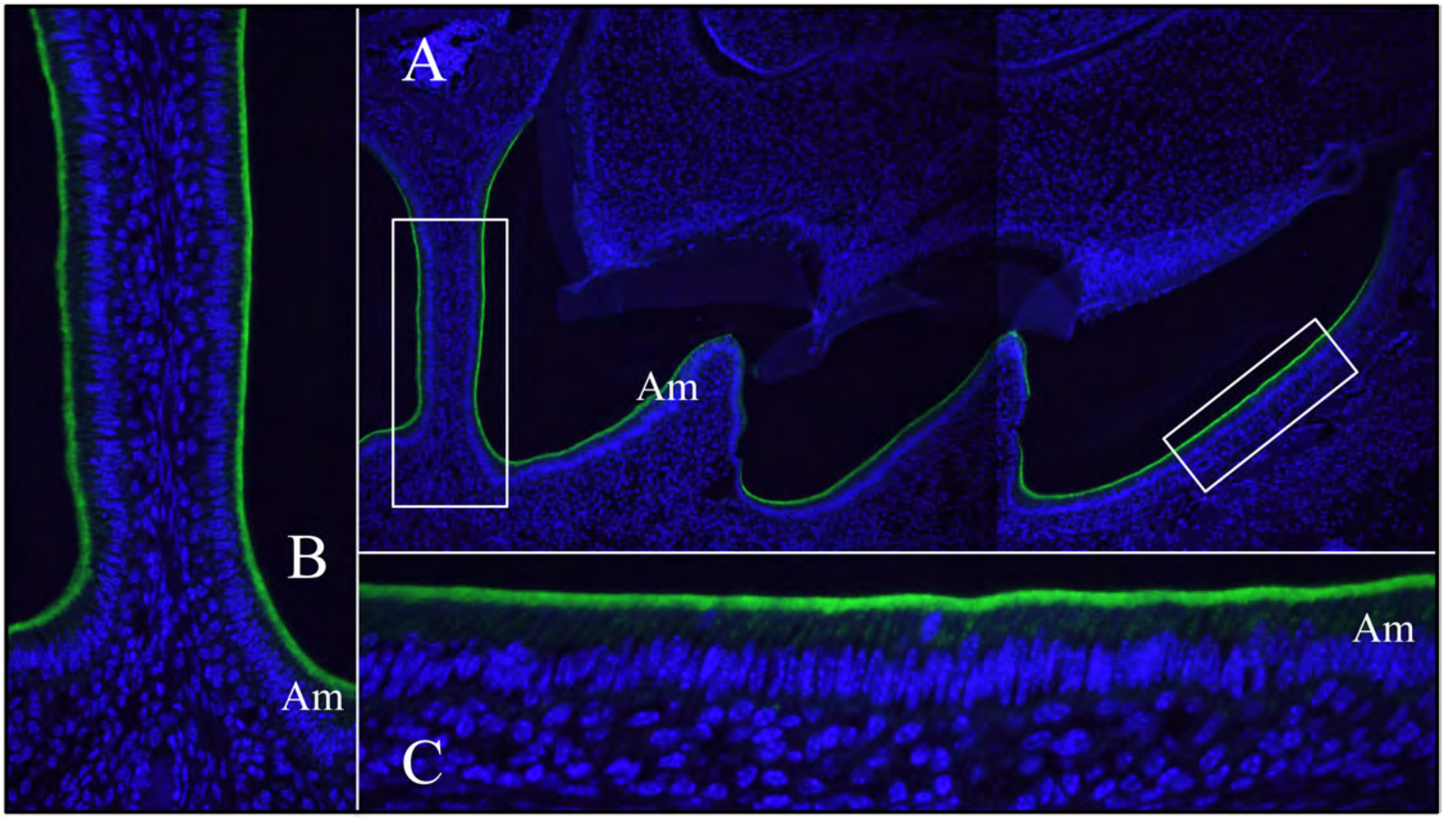


**Figure S12.** Immunohistochemistry of NCKX4 on wild-type D11 maxillary molars. ***A:*** Low-magnification (100×) views of the maxillary first molar and part of the mesial cusp of the second molar. Strong and specific immunoreactivities were detected at maturation stage ameloblasts of both molars. Boxes outline the higher-magnification views in panels B (200×) and C (400×). ***B:*** The interproximal area between the first and second molars at 200×. The signal was highly detected at the distal membranes of ameloblasts. ***C:*** The ameloblasts on the mesial cusp of the first molar at 400×. The short columnar maturation stage ameloblasts exhibited a strong immunoreactivity at the distal membrane and weak in the cytoplasm. ***Key:*** Am, ameloblasts.

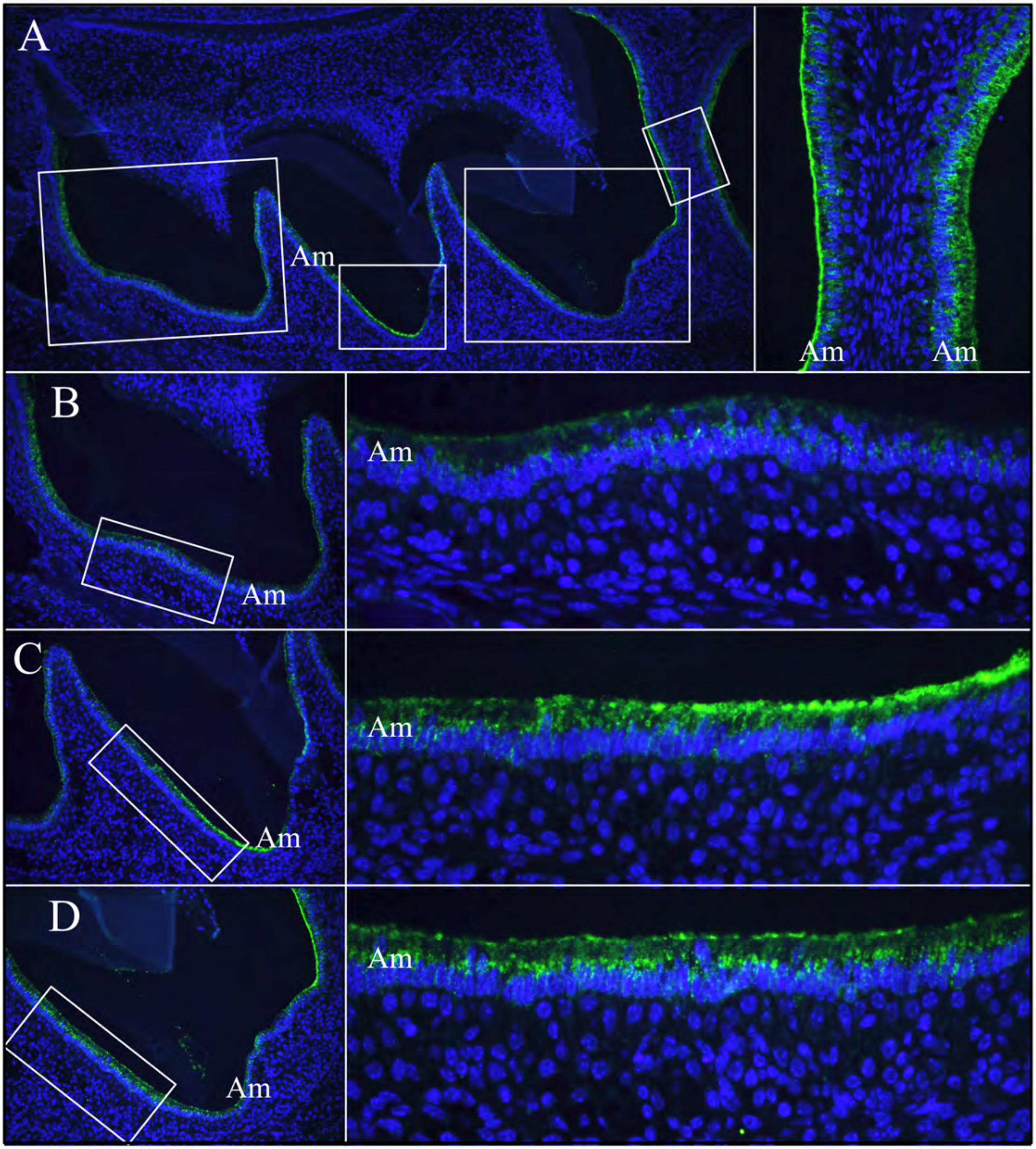


**Figure S13.** Immunohistochemistry of NCKX4 on *Wdr72*$^{-/-}$ D11 maxillary molars. ***A:*** Low-magnification (100×) views of the maxillary first molar and part of the mesial cusp of the second molar. Specific immunoreactivity were detected at maturation stage ameloblasts of both molars. The right panel shows the interproximal area between the first and second molars at 200×. The signal was highly detected at the distal membranes of the first molar ameloblasts, but intracellularly at the second molar ameloblasts. Boxes outline the higher-magnification views in panels B, C, and D. ***B:*** The distal cusp of the first molar at 200×. The box outlines the higher-magnification view (400×) at the right panel. The ameloblasts showed scattered intracellular signals. ***C:*** The middle cusp of the first molar at 200×. The box outlines the higher-magnification view (400×) at the right panel. The ameloblasts at the mesial slope showed strong intracellular immunoreactivities with some signals at the distal membranes. ***D:*** The distal cusp of the first molar at 200×. The box outlines the higher-magnification view (400×) at the right panel. The ameloblasts at the mesial slope exhibited strong intracellular immunoreactivities, particularly localized at the supranuclear area of ameloblasts. ***Key:*** Am, ameloblasts.

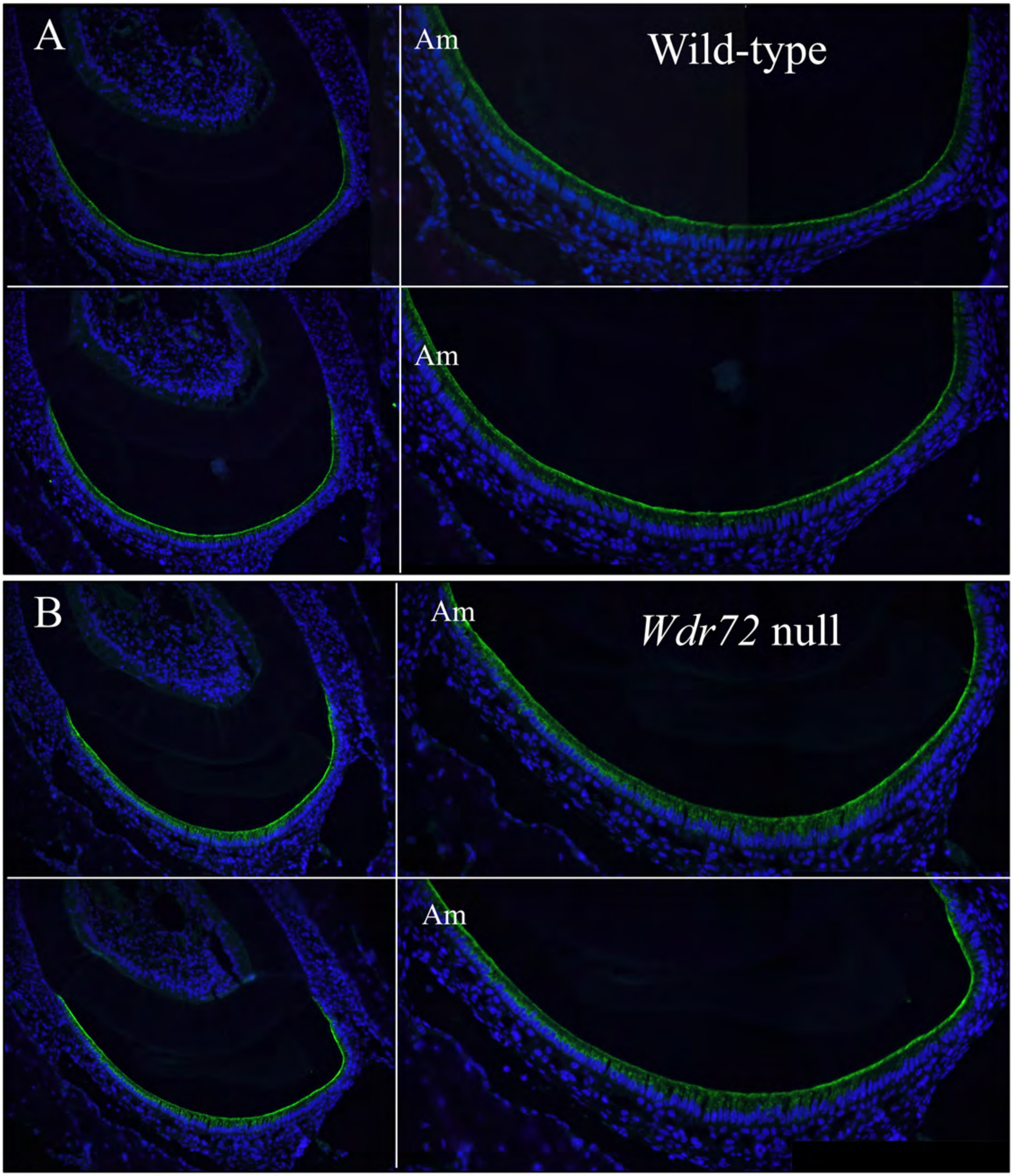


**Figure S14.** Immunohistochemistry of NCKX4 on D14 mandibular incisors. ***A:*** wild-type cross-sections; ***B:*** *Wdr72*$^{-/-}$ cross-sections. Low-magnification (100×) views are on the left; higher magnification (200×) views are on the right. *Wdr72* null mice showed large patches maturation ameloblasts not localizing NCKX4 along their distal membrane. ***Key:*** Am, ameloblasts.

**Table S1.** Mass spectrometry identifications of proteins pulled down with FLAG-tagged recombinant mouse WDR72 expressed in HEK293 cells and immunoprecipitated with anti-FLAG antibody.

| Score | Expectation | Protein ID | Protein Name | MW | % Coverage | Comment | empai |
|---|---|---|---|---|---|---|---|
| 3937 | 0 | 2::sp\|P49792\|RBP2_HUMAN | E3 SUMO-protein ligase RanBP2 OS=Homo sapiens GN=RANBP2 PE=1 SV=2 | 357974 | 41 | | 2.96 |
| 2316 | 0 | 2::sp\|P78527\|PRKDC_HUMAN | DNA-dependent protein kinase catalytic subunit OS=Homo sapiens GN=PRKDC PE=1 SV=3 | 468788 | 22.7 | | 1.08 |
| 1689 | 0 | 2::sp\|P04264\|K2C1_HUMAN | Keratin, type II cytoskeletal 1 OS=Homo sapiens GN=KRT1 PE=1 SV=6 | 65999 | 58.9 | | 12.22 |
| 1199 | 1.80E-115 | 2::sp\|P35527\|K1C9_HUMAN | Keratin, type I cytoskeletal 9 OS=Homo sapiens GN=KRT9 PE=1 SV=3 | 62027 | 54.6 | | 5.89 |
| 821 | 1.20E-77 | 2::sp\|P18583-5\|SON_HUMAN | Isoform D of Protein SON OS=Homo sapiens GN=SON | 266866 | 15.5 | | 0.57 |
| 779 | 1.90E-73 | 1::sp\|Q9ERU9\|RBP2_MOUSE | E3 SUMO-protein ligase RanBP2 OS=Mus musculus GN=Ranbp2 PE=1 SV=2 | 340907 | 12.4 | * * * | 0.41 |
| 726 | 3.20E-68 | 2::sp\|Q9UQ35\|SRRM2_HUMAN | Serine/arginine repetitive matrix protein 2 OS=Homo sapiens GN=SRRM2 PE=1 SV=2 | 299438 | 16.2 | | 0.43 |
| 692 | 8.30E-65 | 2::sp\|P35908\|K22E_HUMAN | Keratin, type II cytoskeletal 2 epidermal OS=Homo sapiens GN=KRT2 PE=1 SV=2 | 65393 | 34 | | 2.09 |
| 679 | 2.00E-63 | 2::sp\|P13645\|K1C10_HUMAN | Keratin, type I cytoskeletal 10 OS=Homo sapiens GN=KRT10 PE=1 SV=6 | 58792 | 34.9 | | 3.78 |
| 604 | 5.50E-56 | 2::sp\|P46013\|KI67_HUMAN | Antigen KI-67 OS=Homo sapiens GN=MKI67 PE=1 SV=2 | 358474 | 18.1 | | 0.48 |
| 481 | 1.20E-43 | 2::sp\|P04259\|K2C6B_HUMAN | Keratin, type II cytoskeletal 6B OS=Homo sapiens GN=KRT6B PE=1 SV=5 | 60030 | 27.8 | | 1.93 |
| 438 | 2.20E-39 | 2::tr\|E7EQV7\|E7EQV7_HUMAN | Keratin, type II cytoskeletal 6C OS=Homo sapiens GN=KRT6C PE=3 SV=2 | 58432 | 29.9 | * * * | 2.02 |
| 380 | 1.50E-33 | 2::sp\|P08779\|K1C16_HUMAN | Keratin, type I cytoskeletal 16 OS=Homo sapiens GN=KRT16 PE=1 SV=4 | 51236 | 36.6 | | 2.21 |
| 341 | 1.10E-29 | 2::sp\|P13647\|K2C5_HUMAN | Keratin, type II cytoskeletal 5 OS=Homo sapiens GN=KRT5 PE=1 SV=3 | 62340 | 27.8 | | 1.43 |
| 326 | 3.50E-28 | 2::sp\|P02533\|K1C14_HUMAN | Keratin, type I cytoskeletal 14 OS=Homo sapiens GN=KRT14 PE=1 SV=4 | 51529 | 37.5 | | 1.67 |
| 163 | 7.10E-12 | 2::sp\|O95613-2\|PCNT_HUMAN | Isoform 2 of Pericentrin OS=Homo sapiens GN=PCNT | 355663 | 5.7 | | 0.12 |
| 155 | 4.10E-11 | 2::tr\|B7WNZ6\|B7WNZ6_HUMAN | Uncharacterized protein OS=Homo sapiens GN=PRRC2C PE=4 SV=2 | 301388 | 6.4 | | 0.15 |
| 95 | 0.00005 | 1::tr\|Q792Z1\|Q792Z1_MOUSE | MCG140784 OS=Mus musculus GN=Try10 PE=2 SV=1 | 26204 | 8.1 | | 0.41 |
| 92 | 0.00009 | 2::sp\|Q86YZ3\|HORN_HUMAN | Hornerin OS=Homo sapiens GN=HRNR PE=1 SV=2 | 282228 | 2.1 | | 0.05 |
| 87 | 0.0003 | 1::tr\|D3YYM4\|D3YYM4_MOUSE | Uncharacterized protein OS=Mus musculus GN=Wdr72 PE=4 SV=1 | 124329 | 3.9 | | 0.12 |
| 82 | 0.00096 | 2::tr\|F8VPF5\|F8VPF5_HUMAN | 5'-AMP-activated protein kinase subunit gamma-1 (Fragment) OS=Homo sapiens GN=PRKAG1 PE=4 SV=1 | 16830 | 11.7 | * * * | 0.7 |
| 82 | 0.00096 | 2::tr\|F5H4D8\|F5H4D8_HUMAN | Ubiquitin OS=Homo sapiens GN=UBC PE=4 SV=1 | 9954 | 28.4 | * * * | 0.55 |
| 81 | 0.0012 | 2::tr\|B4DDT7\|B4DDT7_HUMAN | 5'-AMP-activated protein kinase subunit gamma-1 OS=Homo sapiens GN=PRKAG1 PE=2 SV=1 | 34062 | 9.4 | * * * | 0.5 |
| 79 | 0.0016 | 2::sp\|Q96T58\|MINT_HUMAN | Msx2-interacting protein OS=Homo sapiens GN=SPEN PE=1 SV=1 | 402004 | 3.4 | | 0.07 |
| 74 | 0.0057 | 2::tr\|A5JHP3\|A5JHP3_HUMAN | DCD-1 OS=Homo sapiens GN=DCD PE=2 SV=1 | 12406 | 9.1 | * * * | 0.43 |
| 72 | 0.0097 | 1::tr\|F6XXE6\|F6XXE6_MOUSE | Amylo-1,6-glucosidase, 4-alpha-glucanotransferase (Fragment) OS=Mus musculus GN=Agl PE=4 SV=1 | 93465 | 3.4 | | 0.05 |
| 52 | 0.87 | 2::tr\|D6R991\|D6R991_HUMAN | Matrin-3 (Fragment) OS=Homo sapiens GN=MATR3 PE=4 SV=1 | 48130 | 3.2 | * * * | 0.1 |
| 50 | 1.5 | 1::tr\|E9PXU6\|E9PXU6_MOUSE | Uncharacterized protein OS=Mus musculus GN=Try5 PE=3 SV=1 | 27032 | 7.9 | * * * | 0.18 |
| 48 | 2.1 | 2::tr\|E7EP74\|E7EP74_HUMAN | Golgin subfamily B member 1 OS=Homo sapiens GN=GOLGB1 PE=4 SV=1 | 376907 | 0.8 | | 0.01 |
| 43 | 7.7 | 1::tr\|Q9Z1R9\|Q9Z1R9_MOUSE | MCG124046 OS=Mus musculus GN=Prss1 PE=2 SV=1 | 26118 | 8.1 | * * * | 0.19 |
| 42 | 8 | 2::sp\|P01857\|IGHG1_HUMAN | Ig gamma-1 chain C region OS=Homo sapiens GN=IGHG1 PE=1 SV=1 | 36083 | 4.5 | * * * | 0.14 |
| 37 | 29 | 1::tr\|D3YY41\|D3YY41_MOUSE | Phosphatidylinositol 3-kinase, catalytic, alpha polypeptide, isoform CRA_a OS=Mus musculus GN=Pik3ca PE=4 SV=1 | 110067 | 1.1 | * * * | 0.04 |
| 35 | 44 | 2::sp\|O75969\|AKAP3_HUMAN | A-kinase anchor protein 3 OS=Homo sapiens GN=AKAP3 PE=1 SV=2 | 94691 | 0.8 | * * * | 0.05 |
| 34 | 55 | 1::sp\|A6BLY7\|K1C28_MOUSE | Keratin, type I cytoskeletal 28 OS=Mus musculus GN=Krt28 PE=1 SV=1 | 50315 | 12.6 | * * * | 0.1 |
| 33 | 70 | 2::tr\|Q504X9\|Q504X9_HUMAN | MAP1 light chain LC2 (Fragment) OS=Homo sapiens GN=MAP1A PE=2 SV=1 | 70019 | 2.4 | * * * | 0.07 |
| 32 | 97 | 1::tr\|F7CGG2\|F7CGG2_MOUSE | Transformation/transcription domain-associated protein (Fragment) OS=Mus musculus GN=Trrap PE=4 SV=1 | 405271 | 2.2 | * * * | 0.01 |
| 30 | 150 | 1::tr\|Q9CPN9\|Q9CPN9_MOUSE | RIKEN cDNA 2210010C04, isoform CRA_b OS=Mus musculus GN=2210010C04Rik PE=2 SV=1 | 26405 | 8.1 | * * * | 0.19 |
| 30 | 150 | 2::tr\|H3BVE5\|H3BVE5_HUMAN | Spermatogenesis-associated protein 2-like protein (Fragment) OS=Homo sapiens GN=SPATA2L PE=4 SV=1 | 19865 | 6.7 | * * * | 0.25 |
| 29 | 190 | 2::sp\|O43464-2\|HTRA2_HUMAN | Isoform 2 of Serine protease HTRA2, mitochondrial OS=Homo sapiens GN=HTRA2 | 38469 | 4.2 | * * * | 0.13 |
| 28 | 210 | 2::sp\|Q12830-2\|BPTF_HUMAN | Isoform 2 of Nucleosome-remodeling factor subunit BPTF OS=Homo sapiens GN=BPTF | 324923 | 2.7 | * * * | 0.01 |
| 25 | 460 | 2::tr\|H0YL39\|H0YL39_HUMAN | Cytoplasmic FMR1-interacting protein 1 (Fragment) OS=Homo sapiens GN=CYFIP1 PE=4 SV=1 | 18733 | 4.3 | * * * | 0.27 |
| 24 | 590 | 1::tr\|E9Q309\|E9Q309_MOUSE | Uncharacterized protein OS=Mus musculus GN=Cep350 PE=4 SV=1 | 346244 | 1.5 | * * * | 0.01 |
| 22 | 880 | 2::tr\|B2RP62\|B2RP62_HUMAN | HCG2027369, isoform CRA_a OS=Homo sapiens GN=KIF21B PE=2 SV=1 | 181033 | 2.2 | * * * | 0.03 |
| 21 | 1100 | 1::sp\|Q8C790-2\|CG046_MOUSE | Isoform 2 of Uncharacterized protein C7orf46 homolog OS=Mus musculus | 28317 | 7.8 | * * * | 0.17 |
| 0 | 0 | unassigned | unassigned | | | | |

*** = Tentative 1 significant peptide
